# Transcriptional profiling of single fiber cells in a transgenic paradigm of an inherited childhood cataract reveals absence of molecular heterogeneity

**DOI:** 10.1074/jbc.RA119.008853

**Published:** 2019-06-26

**Authors:** Suraj P. Bhat, Rajendra K. Gangalum, Dongjae Kim, Serghei Mangul, Raj K. Kashyap, Xinkai Zhou, David Elashoff

**Affiliations:** ‡ Stein Eye Institute, Geffen School of Medicine, University of California, Los Angeles, California 90095-7000; § Brain Research Institute, University of California, Los Angeles, California 90095-7000; ¶ Molecular Biology Institute, University of California, Los Angeles, California 90095-7000; ‖ Department of Computer Science and Human Genetics, University of California, Los Angeles, California 90095-7000; ** Department of Medicine, University of California, Los Angeles, California 90095-7000

**Keywords:** crystallin, lens, cataract, vision, transcription, gene expression, crystallin gene expression, genetic cataract, lens, single cells, spatial transcriptomics

## Abstract

Our recent single-cell transcriptomic analysis has demonstrated that heterogeneous transcriptional activity attends molecular transition from the nascent to terminally differentiated fiber cells in the developing mouse lens. To understand the role of transcriptional heterogeneity in terminal differentiation and the functional phenotype (transparency) of this tissue, here we present a single-cell analysis of the developing lens, in a transgenic paradigm of an inherited pathology, known as the lamellar cataract. Cataracts hinder transmission of light into the eye. Lamellar cataract is the most prevalent bilateral childhood cataract. In this disease of early infancy, initially, the opacities remain confined to a few fiber cells, thus presenting an opportunity to investigate early molecular events that lead to cataractogenesis. We used a previously established paradigm that faithfully recapitulates this disease in transgenic mice. About 500 single fiber cells, manually isolated from a 2-day-old transgenic lens were interrogated individually for the expression of all known 17 crystallins and 78 other relevant genes using a Biomark HD (Fluidigm). We find that fiber cells from spatially and developmentally discrete regions of the transgenic (cataract) lens show remarkable absence of the heterogeneity of gene expression. Importantly, the molecular variability of cortical fiber cells, the hallmark of the WT lens, is absent in the transgenic cataract, suggesting absence of specific cell-type(s). Interestingly, we find a repetitive pattern of gene activity in progressive states of differentiation in the transgenic lens. This molecular dysfunction portends pathology much before the physical manifestations of the disease.

## Introduction

**Figure 1.**
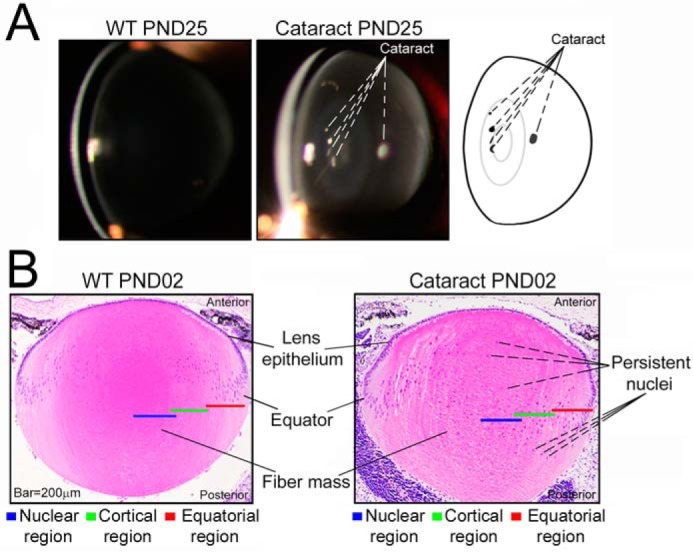
**The transgenic paradigm for the inherited cataract in the mouse lens.** *A,* slit lamp images of WT PND25 and mutant transgenic (cataract PND25) lenses (19). The schematic on the *extreme right* shows locations of the opacities (cataract). *B,* paraffin sections (4 μm) of the 2-day-old WT PND02 and transgenic (cataract PND02) lenses stained with hematoxylin (nuclei, *blue*) and eosin (cytoplasm, *pink*). Note persistent and displaced nuclei (*blue*) all through the fiber mass in the transgenic, cataract PND02 lens. In the WT lens, the nuclei are only seen in the differentiating fiber cells and not throughout the lens (19). The anterior epithelial cells differentiate at the equator and add to the fiber mass. The *red*, *green*, and *blue bars* (200 μm each) indicate the spatial locations named equatorial, cortical, and nuclear regions, respectively.

The functional phenotype of the ocular lens is transmission of focused light into the eye. This physical phenotype is the culmination of a coordinated orchestration of gene activity and morphogenesis, which in the adult lens commences in the edges (equator) of the epithelium that covers the anterior face of the lens (1) (see Fig. 1). Differentiation produces long, elongated fiber cells, which are layered one over the other in an ordered and temporally regulated fashion. The youngest fiber cells, therefore, are on the outside, whereas the oldest, terminally differentiated cells are inside, in the center of the lens. The fiber cells thus constitute >95% of the volume of the adult lens, in which the oldest terminally differentiated fiber cells, in the middle, make the visual axis. Notably, the differentiation of fiber cells, from the epithelial cells, is attended by high expression of crystallins, proteins that generate transparency (2–6).

Recently, we developed a method to isolate single fiber cells from a 2-day-old postnatal lens (PND02),^2^ in a fashion that allows retention of their spatial context. Analysis of these fiber cells, representing different regions/states of differentiation, revealed that the cortical fiber cells that connect the equatorial and the nuclear regions were transcriptionally highly variable. Importantly, a specific group of crystallin genes show coordinated increase in their expression in the fiber cells derived from the nuclear region of the lens (7). This new knowledge, revealing deterministic expression of crystallin genes, following highly variable gene activity in the cortical fiber cells, indicated that transcriptional heterogeneity might be an important contributor to a progression toward terminal differentiation and transparency in the ocular lens.

Analysis of the gene expression heterogeneity in single cells in multiple paradigms has led to the identification of a number of new cell-types in many eukaryotic systems (8–12). However, although the recognition of new cell types illuminates the cellular complexity that makes a tissue, the role of transcriptional heterogeneity, if any, remains conjectural (13–15). It is unclear whether molecular heterogeneity at the single cell level is relevant to the realization of the functional phenotype of a tissue. Here we present our findings, working with a transgenic paradigm of an inherited childhood cataract to address this question.

Cataract is a disease of the ocular lens where the fiber cells, instead of remaining transparent, become opaque, which inhibits light transmission. Because, in the ocular lens, the functional phenotype of fiber cells is transparency and the disease phenotype is a cataract (opacification), it would be meaningful to examine the status of transcriptional heterogeneity (which is characteristic of the WT transparent fiber cells) (7) in the individual fiber cells of a cataract (lens).

For these investigations, we have used the transgenic model of a genetic cataract known as the lamellar cataract. Lamellar cataract is the most prevalent inherited childhood cataract (16, 17); it is associated with mutations in the DNA-binding domain of the heat-shock transcription factor, *HSF4* (18). We have previously generated transgenic mouse models for this disease by introduction of disrupted or mutated *Hsf4* gene sequences within a bacterial artificial chromosome (19, 20). The early childhood cataract phenotype is faithfully recapitulated in these transgenic models, both spatially as well as temporally (19, 20). The disease appears in the postnatal lens as cataracts (opacities) in a spatially restricted area in a single or a few fiber cells (or lamellae) (Fig. 1) (19).

Here we investigate this unique paradigm at the single-cell level in a developing mouse lens genetically predisposed to cataractogenesis (Fig. 1*B*). We worked with the postnatal 2-day-old (PND02) transgenic mutant lens, which allows us to probe early molecular changes, much before the cataract pathology is visible after the eyes open (Fig. 1*A*).

## Results

Fig. 1*A* shows the lens at postnatal day 25 (PND25) where the cataracts are visible. The transgenic lens looks like a normal WT lens in its overall morphology. The pathology (cataract) is visible only in a few fiber cells (Fig. 1*A*, *schematic*) whereas the rest of the lens looks normal. Although the mutant lens physiognomy looks normal, a cross section of the lens at PND02 reveals the persistence of nuclei (Fig. 1*B*, cataract PND02), an abnormal phenotype, known to be associated with the loss of *Hsf4* gene activity (21) (19); the nuclei are seen in the anterior as well as in the posterior of the lens (Fig. 1*B*, cataract PND02) (19). Note that in the WT lens, the fiber cell nuclei are organized in a “bow” at the equator (Fig. 1*B*, WT PND02).

About 500 fiber cells, from five different transgenic lenses were manually isolated following the procedures established previously (7). These fiber cells represent three different regions and, therefore, three different states of differentiation (equatorial, cortical, and nuclear, indicated by *red*, *green*, and *blue bars*, respectively, in Fig. 1*B*). Each fiber cell was interrogated for the expression of 95 genes (including 17 crystallins and 78 noncrystallins; Table S1), just as was done previously with the WT lens fiber cells (7).

### Unsupervised clustering shows absence of transcriptional heterogeneity

**Figure 2.**
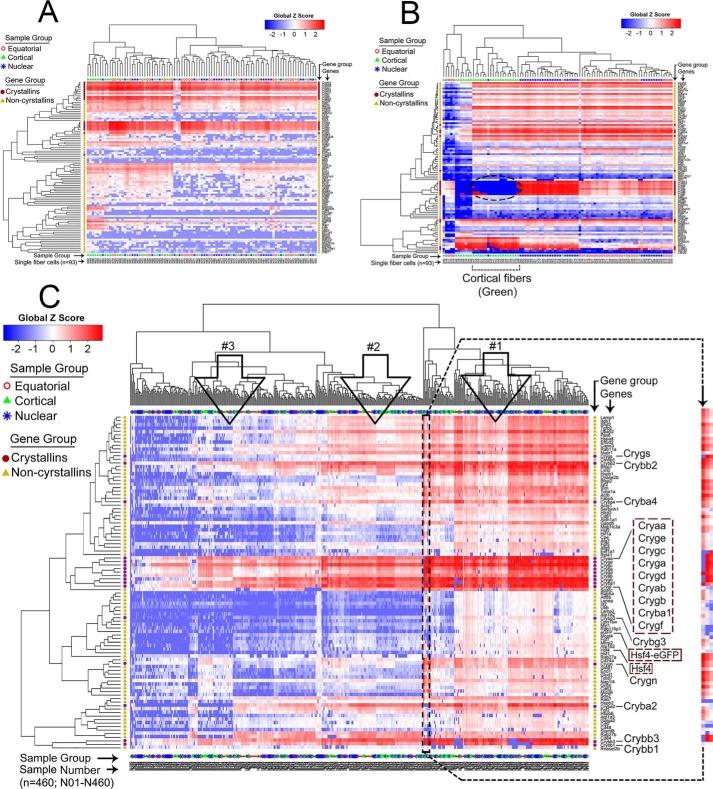
**Unsupervised clustering reveals general lack of heterogeneity in fiber cells isolated from different regions of the mutant transgenic lens.** *A*, hierarchical clustering of data obtained from 93 fiber cells (interrogated for 95 genes) from three regions (equatorial = 30, cortical = 30, nuclear = 33) of a PND02 transgenic mutant lens. *B*, hierarchical clustering of the data obtained from 93 fiber cells (interrogated for 94 genes), isolated as in (*A*) from the three regions of a PND02 WT lens (equatorial = 30, cortical = 30, nuclear = 33). The *dotted oval* indicates the cortical fiber cells (*green*), which show poor crystallin and high noncrystallin expression. This group of fiber cells is not seen in the transgenic (cataract) lens fiber cells. *A* and *B* present a comparative picture in single lenses. A complete analysis of the WT lens fiber cells (based on data from five different lenses) is published in Ref. 7. The data in (*B*) are an independent determination, not from one of the WT lenses presented in Ref. 7. *C,* 460 single fiber cells, isolated from five different transgenic lenses, were interrogated with 95 genes (17 crystallins (●) and 78 noncrystallins (▴) (shown on *y* axis, all crystallins and *Hsf4-eGFP* and *Hsf4* are shown in a larger font). These data are comparable to WT data in Ref. 7. Here, three dominant patterns emerge, fiber cells with high expression (*open arrow 1*), fiber cells with low expression (*open arrow* 3), and fiber cells in-between (*open arrow 2*). The expression of the endogenous *Hsf4* and the *Hsf4-eGFP* transgene (19) is seen in most fiber cells under *arrow 1*. These fiber cells express a large number of crystallin genes (*dotted violet rectangle on the right*) as well as a number of noncrystallins at high levels. A fiber cell, at the boundary of *arrow 1* and *arrow 2*, is demarcated and is shown magnified on the *right*. This is sample N283; it is a fiber cell derived from the cortical region (see Fig. S2). This fiber cell shows poor crystallin expression but high expression of noncrystallins. These cells are seen in the WT lens in significant numbers (∼29% of all cortical fibers) but are not found here in the mutant transgenic lens, except this lone cell. Note that the order of the crystallin cluster in *A* is different from the cluster in *C* (the euclidean distances change with the number of samples). *Scale bar* with *Z* scores, −2 to +2 (*red* = high expression, *blue* = low expression). Alphanumeric values N1–N460 on *x* axis represent sample groups (fiber cells from three regions: *open red circles* = equatorial region (*n* = 150); *green triangles* = cortical region (*n* = 150); *blue asterisks* = nuclear region (*n* = 160)). The data presented in *A* are an independent determination and are not a part of the data in *C*.

In the WT lens, the transcriptional landscape of the fiber cells keeps changing from one region to the other as differentiation proceeds (3–6, 22). Our recent work with single fiber cells isolated from three different regions of the WT PND02 mouse lens revealed the existence of a population of cortical fiber cells, which show very poor expression of crystallins but high expression of noncrystallin genes (7). These cells contribute >80% of the transcriptional variability in isolated single fiber cells of the WT lens. Fig. 2, *A* and *B* show unsupervised clustering of the gene expression data obtained from single fiber cells, isolated from one transgenic lamellar cataract lens and one WT lens, respectively. It is evident that a group of cortical fibers which show poor crystallin expression but high noncrystallin expression (Fig. 2*B*, *dotted oval*) are absent in the transgenic lens (Fig. 2*A*).

Fig. 2*C* shows a cumulative analysis of the data obtained from five lenses (460 fiber cells in all, 92 fiber cells from each lens), confirming what we see in the data derived from one lens (Fig. 2*A*). In fact, the data presented in Fig. 2*C* for the transgenic lens are directly comparable with the data published for the five WT lenses (7). In Fig. 2*C*, we can recognize three predominant groups of cells that express crystallins and noncrystallins at variable levels (*arrowheads* 1–3). There is a group of fiber cells, which express crystallins as well as the noncrystallins at high levels (*arrowhead* 1); a group of fiber cells at an intermediate level (*arrowhead* 2); and a group of fiber cells which show poor expression of most of the genes (*extreme left columns* of Fig. 2*C*, *arrowhead 3*). The expression pattern in Fig. 2*C* suggests general uniformity with respect to the expression of genes all across the 460 fiber cells; we do not see fiber cell(s) with specific expression of a gene(s). This is a remarkable departure from the data obtained previously with the WT lens fiber cells, where various fiber cells can be characterized based on the expression of a specific gene(s) (7).

A closer examination of the data in Fig. 2*C* shows a fiber cell with very low crystallin expression and high noncrystallin expression (*magnified image on the right*). We traced this fiber cell to the data from cortical fiber cells (Fig. S2), which suggests that this is a rare normal fiber cell in the mutant lens. In fact, in the WT lens 29% of the cortical fiber cells show low crystallin but high noncrystallin gene expression (7). This group of cells is absent in the transgenic lens fiber cells (Fig. 2, *A* and *C*).

### PCA sifts 460 fiber cells into three clusters of spatially mixed populations of cells

**Figure 3.**
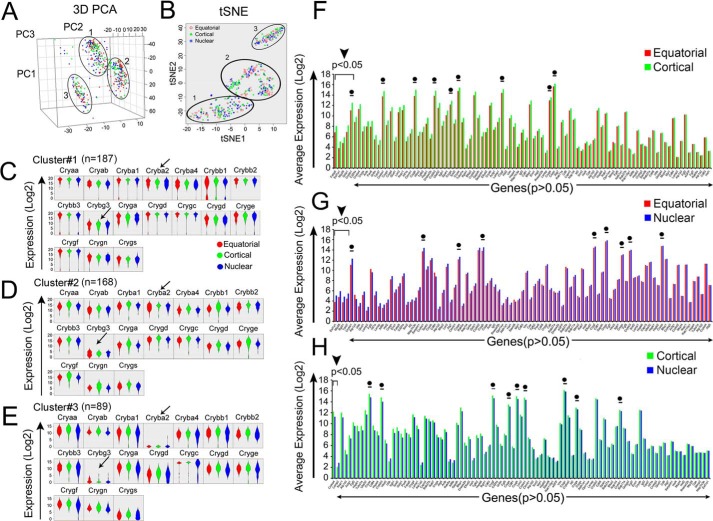
**PCA sifts 460 single fibers into three clusters of mixed populations of cells.** *A* and *B,* the PCA (a standard linear dimensional reduction method) and tSNE (a nonlinear relationship in the data set based on the probability distribution), respectively, generate three distinct sample clusters. The PCA cluster 1 (*n* = 187), cluster 2 (*n* = 168), and cluster 3 (*n* = 89) all contain mixed populations of cells, *red*, *green*, and *blue*). *C–E*, gene expression distributions (violin plots) within these three clusters; only data for crystallin genes are shown (complete data for all 95 genes are presented in Fig. S3). All the clusters show high crystallin expression. In cluster 3, Cryba2 and Crybg3 are significantly low (*arrows*). Fig. S3*D* contains the PCA loading plots to provide information about genes that may make these clusters. *F–H*, comparative average expression of 95 genes in different regions of the transgenic lens (see also ANOVA analysis presented in Fig. 4 and volcano plots in Fig. S4). *Bar graphs* represent average expression (log2) on the *y* axis; the genes are arranged from low to high *p* value on the *x* axis. *F*, equatorial (*red*) *versus* cortical (*green*) fiber cells. Only five genes (Fabp5, Eef1a1, Gstp1, Mip26, and Crybb1) are significantly different (<0.05) (*black arrowhead*, *top left*). *G*, equatorial (*red*) *versus* nuclear (*blue*) region show four differentially regulated genes (Eef1a1, Mip26, Rpl41, and Tdrd7) with *p* < 0.05 (*black arrowhead*, *top left*). *H*, cortical (*green*) *versus* nuclear (*blue*) comparison shows only one (Cryba4) differentially expressed gene (*black arrowhead*, *top left*). (●) = crystallin genes known to be coordinately expressed in the WT fiber cells (7).

Principal component analysis (PCA) of the transcriptional activity of 95 genes in 460 fiber cells of the transgenic lens shows three top clusters (Fig. 3*A*). Notably, all of the clusters are composed of mixed populations of cells (*red*, *green*, and *blue* representing equatorial, cortical, and nuclear fiber cells, respectively). Similar analysis in the WT fiber cells produced five clusters. Of these, there were at least two clusters of fiber cells which were predominantly composed of cells spatially traceable to either cortical or nuclear regions (7). Analysis of the data (Fig. 3*A*) from the mutant (transgenic) fiber cells reveals that there are no clusters which represent a specific region or an absence or presence of one gene activity. Cluster 1 (*n* = 187 cells) generates >88% variation in these data and is composed of 58, 58, and 71 fiber cells from the equatorial, cortical, and nuclear regions, respectively. The other two clusters are not very different in their spatial derivations.

Fig. 3, *C–E* presents violin plots (probabilistic distributions of various populations of cells based on expression) of all 17 crystallins (the data for all 95 genes are presented in Fig. S3, *A–C*). All three clusters contain cells which express most crystallins at high levels (Fig. 3, *C–E*). There are differences, however: Cluster 3 differs from the first two clusters in that it shows low expression of Cryba2 and Crybg3 (Fig. 3*E*, *arrows*). The presence of most crystallin transcripts at high concentrations in all clusters suggests unusual uniformity of crystallin expression (PCA loading plots are presented in Fig. S3, *D* and *E*).

Fig. 3, *F–H* presents a gene-by-gene comparison of the expression profile of all 95 genes in the fiber cells from different regions of the transgenic lens (equatorial *versus* cortical, equatorial *versus* nuclear, and cortical *versus* nuclear, respectively). These data demonstrate similar gene activities in the fiber cells from the equatorial, cortical, and nuclear regions establishing the absence of molecular transitions between different regions in the transgenic lens.

**Figure 4.**
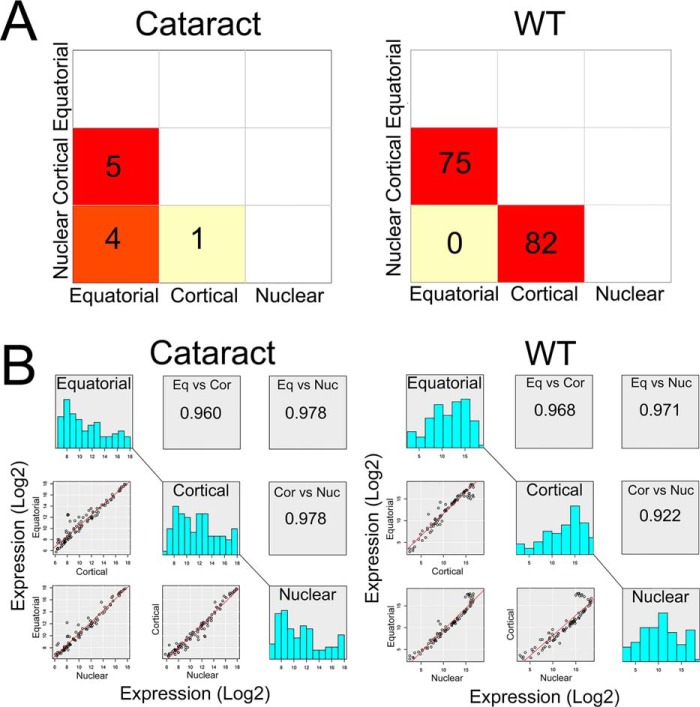
**There is little differential gene activity between fiber cells of different regions in the mutant lens.** *A*, the matrix square plot (3 × 3) represents pairwise ANOVA summaries of 95 and 94 genes in cataract and WT, respectively. In the cataract, fiber cells from the equatorial *versus* cortical region show five differentially expressed genes (*red square, middle left*), equatorial *versus* nuclear region shows four differentially expressed genes (*red square, bottom left*), and cortical *versus* nuclear region shows just one gene, which is differentially expressed (*light yellow square*). The corresponding respective WT comparisons are 75, 0, and 82. *B*, pairwise comparisons of scatter plot of genes (*bottom left* for each, cataract and WT) and their average expression (log2) and binned (*bar graphs*) for three regions, connected diagonally by a *line*. The Pearson correlation coefficients between the comparisons of three sample groups are shown on the top right. For cataract fiber cells, equatorial *versus* cortical region, it is 0.960; for equatorial *versus* nuclear region, it is 0.978; and for the cortical *versus* nuclear region, it is 0.978. The WT correlation coefficients with the same comparisons are 0.968, 0.971, and 0.922, respectively. Note the significant difference in the transition between the cortical and the nuclear fiber cells between the cataract (0.978) and the WT (0.922). For this analysis, the WT data are from Ref. 7.

Analysis of differential gene activity in various regions of the WT transparent lens reveals that of the 94 genes, there are 75 genes differentially expressed between the equatorial and the cortical fibers and 82 genes between the cortical and the nuclear fibers (Fig. 4*A*, WT). In comparison, in the fiber cells from the transgenic lens (Fig. 4*A*, cataract), the corresponding numbers are 5 and 1, respectively. This is further corroborated by scatter plots presented in Fig. 4*B* (see also volcano plots in Fig. S4, showing absence of differential gene activity).

These gene expression data are also supported by the similarity of the violin plots and the corresponding box plots of gene expression in fiber cells from all three regions, suggesting that there are similar cell populations or cell-types all across the developing transgenic lens (Fig. S5, *A–C*).

### Lack of variability of gene expression in cortical fiber cells

**Figure 5.**
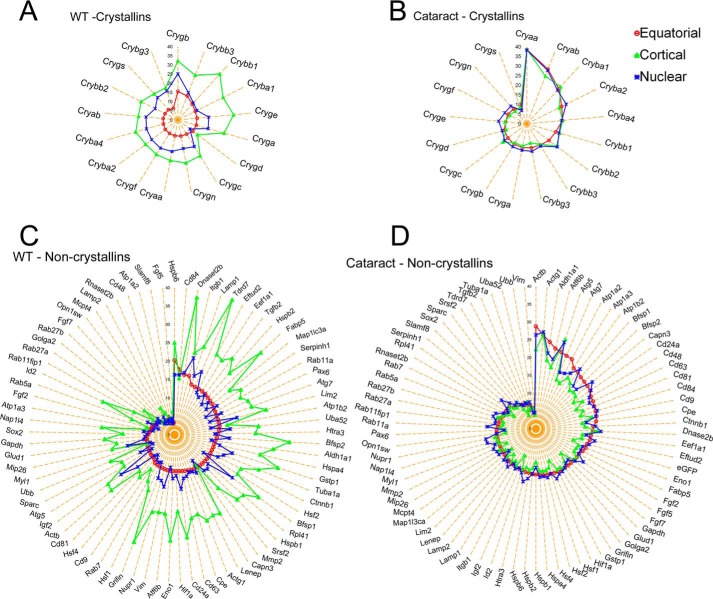
**Absence of gene expression variance in fiber cells in the mutant transgenic lens.** *A* and *C*, radar plots show variance of gene expression in crystallins and noncrystallins, respectively, in the WT lens. The single fiber cells in the cortical region (*green line with triangles*) show significantly high variance compared with equatorial (*red*) and nuclear fiber (*blue*) cells. *B* and *D*, variance of crystallin and noncrystallin gene expression, respectively, in fiber cells from the transgenic (cataract) lens. There is very little variance seen in the expression levels between the fiber cells from the equatorial (*red*), cortical (*green*), and nuclear (*blue*) regions. The data for WT analysis were taken from Ref. 7.

One of the landmarks of the developing WT lens is the high transcriptional heterogeneity in the fiber cells derived from the cortex (7). It is in this context, therefore, that the absence of variation in the expression of crystallin and noncrystallin genes in the fiber cells of the transgenic lens is striking (Fig. 5, *B* and *D*). The WT lens cortical fiber cells show highest variation, both in crystallins as well as in noncrystallin gene expression (Fig. 5, *A* and *C*, *green*). These data augment the apparent uniformity of cellular population distributions seen above in the PCA clusters and lack of differential gene activity (Figs. 3, *F–H*, and 4).

There is appreciable increase in the noncrystallin gene activity in the cortical fiber cells in the WT lens; this activity obviously is highly variable (Fig. 5*C* and Ref. 7). Comparison of the noncrystallin activity in the WT and transgenic lens, however, reveals that decrease in the variability of expression in the cortical fiber cells in the transgenic lens is also accompanied by quantitatively lower transcriptional activity (Fig. 5, compare *C* and *D*).

### Repetitive gene activity in fiber cells of different regions in the transgenic (cataract) lens

**Figure 6.**
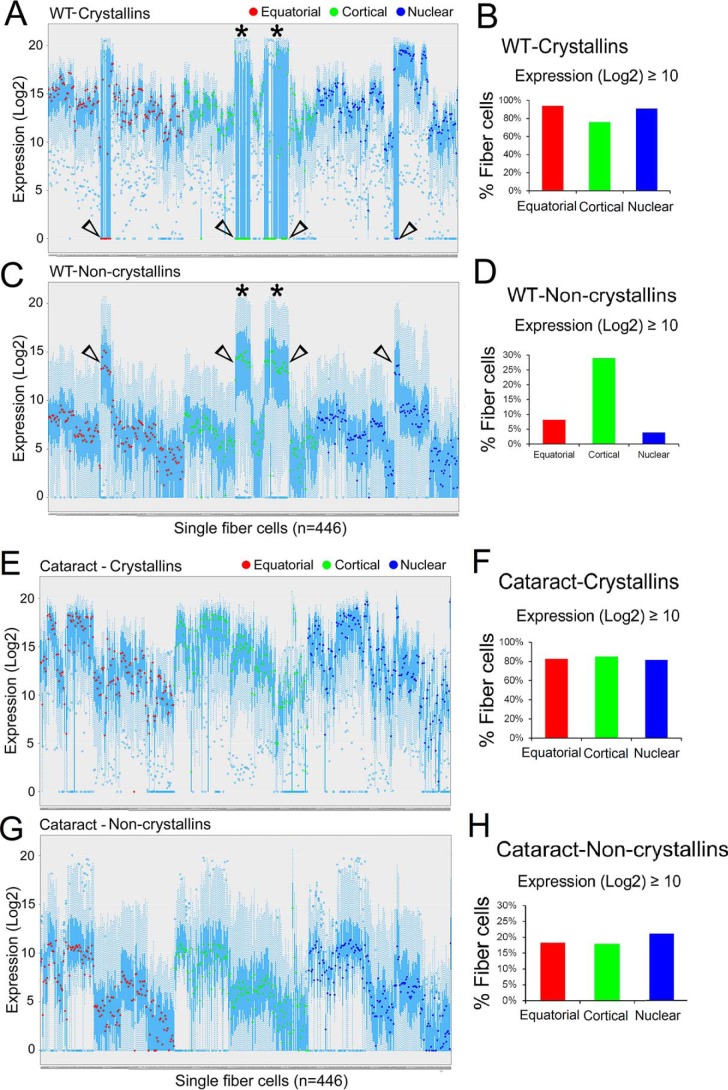
**Repetitious gene activity in single fiber cells derived from different regions of the transgenic (cataract) lens.** Box-and-whisker plots of gene expression are presented. Each box plot represents a single fiber cell. The *red* (equatorial), *green* (cortical), and *blue* (nuclear) *dots* are the medians of expression (log2) in individual fiber cells. *Y*-axis = expression (log2); *x* axis = fiber cells. *A*, box-and-whisker plots of the gene expression of 17 crystallin genes in 446 WT fiber cells. *Asterisks* show cortical fiber cells with low crystallin expression (*arrowheads*). *B*, bar graphs of the percentages of fiber cells expressing crystallins with expression log2 above ≥10 in *A*. In the WT, 75.86% of cortical fiber cells show log2 expression ≥ 10 compared with 93.87% in the equatorial and 90.90% in the nuclear fibers. *C*, expression profiles of 77 noncrystallin genes in 446 WT fiber cells. *Asterisks* show cortical fiber cells with high noncrystallin expression (*arrowheads*). *D*, bar graphs of the data in *C*. The noncrystallin gene expression (log2) ≥ 10 is significantly (*p* < 0.05) higher in cortical fiber cells (28.96%) compared with equatorial (8.16%) and nuclear (3.89%) fiber cells. None of this variation is seen in transgenic fiber cells (see *G* and *H*). *E*, gene expression profile of 17 crystallin genes in 446 fiber cells isolated from the lamellar cataract transgenic lens. *F,* bar graphs of the percentages of fiber cells with expression log2 ≥ 10 in *E*. Fiber cells in all regions express same level of crystallins (compare with *B*). *G*, expression of 78 noncrystallin genes in 446 single fiber cells in transgenic (cataract) lens. *H,* bar graphs of expression in *G*. Note very little change in noncrystallin expression (compare with *D*). Note that spikes of low crystallin expression (*arrowheads* in *A*) and high noncrystallin gene expression (*arrowheads* in *C*) interrupt the patterns of expression in the WT lens. These are missing from the transgenic (cataract) fiber cells (*E* and *G*). Interestingly, the gene activity in *E* and *G* presents a repetitive pattern. WT data are from Ref. 7.

Fig. 6 presents a panoramic view of the 95 gene activities across the landscape of individual fiber cells in the WT PND02 lens (Fig. 6, *A* and *C*) and in the transgenic (cataract) lens (Fig. 6, *E* and *G*). In the WT, these data reveal a pattern that is interrupted by large variations in gene activity, be it the crystallins (Fig. 6*A*, *open arrowheads*) or the noncrystallins (Fig. 6*C*, *open arrowheads*). Note that in Fig. 6*A*, the medians (*red, green*, and *blue dots*), which are close to *x* axis (*open arrowheads*) indicate poor/absence of crystallin expression. The same fiber cells, however, show high noncrystallin gene expression (Fig. 6*C*, *open arrowheads*). Note that in the WT, about 75% of the cortical fiber cells (*green*) express crystallins at (log2) ≥ 10 (Fig. 6*B*). In the same region about 29% of cells show high noncrystallin expression (log2) ≥ 10 whereas the equatorial and nuclear fiber cells (*red* and *blue bars*, respectively) show much lower levels (Fig. 6*D*). These large variations in expression from region to region are absent in the fiber cells of the transgenic (cataract) lens (Fig. 6, *F* and *H*). Interestingly, however, the transgenic fiber cells show a repetitive pattern of gene activity, both among crystallins (Fig. 6*E*) as well as in noncrystallins (Fig. 6*G*).

### Uniform crystallin expression in fiber cells from different regions of the transgenic lens

**Figure 7.**
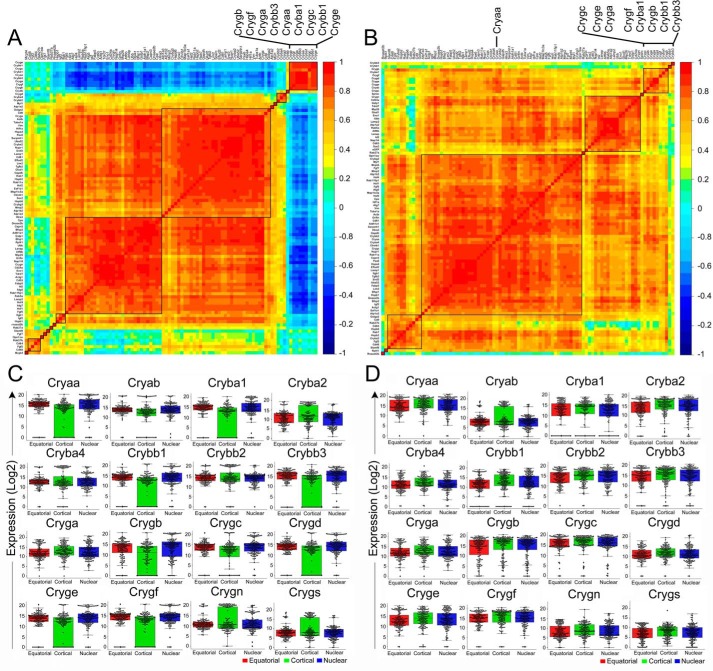
**Absence of the variability of crystallin gene expression in the transgenic lens.** *A* and *B*, Pearson correlation coefficient plots for the WT (*A*) and cataract (*B*) genes. The data represent a range of correlation from −1 to +1 (color scale on right of each plot). 0 = no correlation, +1 (*red*) = positive linear correlation, and −1 (*dark blue*) = negative linear correlation between the genes. *A,* correlation matrix plot of WT lens fiber cells (probed for 94 genes including 17 crystallin and 77 noncrystallin genes). Note that a discrete set of nine crystallin genes (Cryga, Crygf, Cryga, Crybb3, Cryaa, Cryba1, Crygc, Crybb1, and Cryge) (*indicated on the top right corner*) show positive correlation (*red*) within the group and negative correlation with all other genes (*sky blue* to *dark blue*). Multiple other gene correlations are evident, including the noncrystallin gene activity (*two red squares*). *B*, the correlation matrix plot of the transgenic (cataract) fiber cells (probed for 95 genes including17 crystallins and 78 noncrystallin genes). Most genes here show homogenous distribution in the matrix plot (*a red square in the middle*). Note the nine crystallins (shown in *A*, *top right corner*) are not as tightly coordinated. *C*, box and swarm plots of crystallin gene expression in the WT fiber cells. Note the variability in the crystallins in cortical fiber cells (*green boxes*). A number of these gene activities (Cryaa, Cryba1, Crybb1, Crybb3, Cryga, Crygb, Crygc, Cryge, and Crygf) are coordinately regulated (see *A*) and characteristic of the terminally differentiated nuclear fiber cells (7). *D*, box and swarm plots of crystallin gene expression in the transgenic (cataract) fiber cells show mostly compact boxes indicating absence of variability. Box plots for all noncrystallin genes are presented in Fig. S6. For this analysis, WT data were from Ref. 7.

The repetitive patterns of gene activity result in the absence of finding specific gene activities that may be coordinated and/or represent specific fiber cells or regions within the developing lens. For example, the data in Fig. 7, *A* and *B* shows Pearson correlation plots of gene expression in the WT (Fig. 7*A*) and in the transgenic (cataract) fiber cells (Fig. 7*B*). Although everything seems to be uniformly correlated in the cataract fiber cells (Fig. 7*B*), there are specific correlations evident in the WT fiber cells, the prominent one being the nine crystallin genes (Fig. 7*A*, *top right*) and their inverse correlation with the noncrystallin activity in the rest of the fiber cells (Fig. 7*A*, *blue*). Note that in the fiber cells of the transgenic cataract, Cryaa and the eight crystallins are not as coordinated (Fig. 7*B*, *top right*) as in the WT (Fig. 7*A*). The data in Fig. 7, *A* and *B* represent all genes in about 460 fiber cells of the WT and the transgenic lens, respectively.

In Fig. 7, *C* and *D*, we compare only the expression of crystallin genes in the WT and transgenic fiber cells. In the WT lens (Fig. 7*C*), the expression of crystallins in cortical fiber cells (*green*) is highly variable (mostly in the lower quartile, whereas the median is skewed up, *green boxes*. The expression profiles in the fiber cells of the transgenic lens (Fig. 7*D*) from all regions (*red*, *green*, and *blue*) are uniform and with rather compact distributions indicating absence of heterogeneity. The variability of noncrystallin genes is also high in the cortical fiber cells in the WT than in the transgenic lens (see box plots presented in Fig. S6).

**Figure 8.**
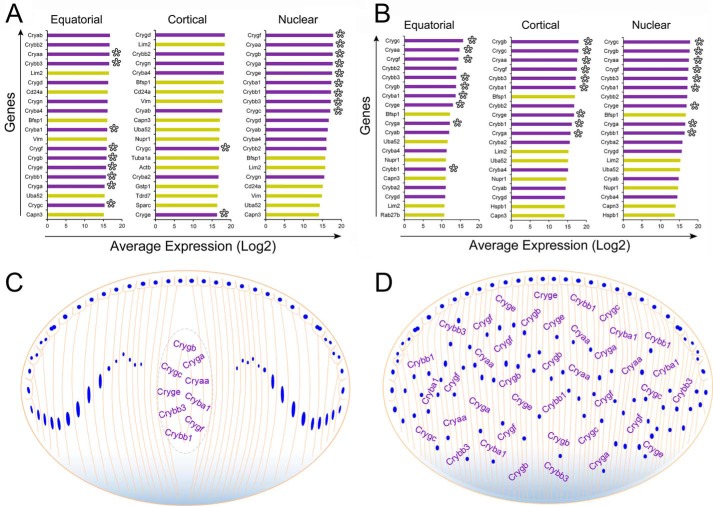
**Uniformity in the expression of crystallin genes in the transgenic (cataract) lens.** *A,* top 20 expressed genes in single fiber cells from the equatorial, cortical, and nuclear regions of the WT lens. *X*-axis = average expression (log2); *y* axis = genes. There are 14 crystallins and 6 noncrystallins in the top 20 in the equatorial and nuclear fiber cells. In the cortical fiber cells, the top 20 are represented by only 8 crystallins and 12 noncrystallins. Of the 8 crystallins here, however, there are only 2 crystallins which are characteristic of the terminally differentiated nuclear fiber cells (*asterisks*). *B*, top 20 expressed genes in single fiber cells in the equatorial, cortical, and nuclear regions of the transgenic (cataract) lens. Note that all the 20 genes (14 crystallins and 6 noncrystallins) are expressed in all the fiber cells irrespective of their spatial derivation. This is in stark contrast to the WT lens where the cortical region fibers present a very different pattern. Crystallins = *purple bars*, noncrystallins = *golden bars. C,* a schematic of the lens showing spatially relevant expression of 9 crystallins coordinately expressed in the terminally differentiated fiber cells of the lens nucleus, the future visual axis of the lens. Of the 9 crystallins 5 belong to the γ-crystallin family, which are known to be proteins with highest refractive index complement (43–45). This does not mean that there are no crystallins in the rest of the lens. This representation is made based on the data in *A* and data that show increased expression of these crystallins in the nuclear fiber cells upon transition from the cortical fiber cells (7) and their negative correlation with noncrystallin activities (Fig. 7*A*). *D*, a schematic of the postnatal day 2 mouse lens with the spatially misplaced (irrelevant) distribution of the expression of nine crystallin genes in all fiber cells in all regions of the developing lens. WT data for this analysis is taken from Ref. 7.

The special status of the cortical fiber cells is even more evident in light of the observation that the expression of nine crystallins (Cryaa, Crybb3, Cryba1, Crygf, Crygb, Cryge, Crybb1, Cryga, and Crygc) associated with terminal differentiation is highly coordinated (7) (Fig. 7*A*). When assessing the top 20 gene activities in the fiber cells representing three lens regions (Fig. 8, *A* and *B*), these nine crystallin genes make the list of top 20 genes only in the equatorial and nuclear fiber cells of the WT lens (Fig. 8*A*, *asterisks*). Importantly, only two of the nine crystallins (Fig. 7A) are seen in the top 20 genes expressed in these fiber cells (Fig. 8*A*, cortical, *asterisks*). In comparison, the pattern of top 20 genes in all three regions of the transgenic fiber cells is very similar if not identical (Fig. 8*B*); noticeably, here 14 crystallins are expressed in all the regions, including the 9 (*asterisks*) associated with terminal differentiation (Fig. 7*A*) (7). These data suggest that crystallin expression alone does not guarantee functional differentiation.

## Discussion

The seminal observation made in this investigation is that the molecular heterogeneity, characteristic of the fiber cells in the developing WT lens is absent in the transgenic paradigm of an inherited cataract (Figs. 2–8).

Molecular and morphological heterogeneity is a highly significant facet of organ development. A nascent cell may go through a number of cell states and/or cell-types before reaching the terminally differentiated functional state. Recent technological capabilities to assess gene expression have revealed that the molecular landscape of single cells as assessed by transcriptional profiling is highly variable (8, 10, 11, 23, 24). How this heterogeneity relates to the functional phenotype within a tissue remains to be understood (13–15, 25). The developing ocular lens presents a simple and a developmentally well-defined paradigm for following the molecular progression of differentiation, starting from a nascent epithelial cell in the surface (equator) to a terminally differentiated fiber cell in the center (nucleus) of the lens. What is remarkable though is that each one of these cell-states is accessible. Having established the initial parameters of the status of transcriptional heterogeneity at the single fiber cell level in the WT lens, we investigated a lens genetically (developmentally) predisposed to a pathology (lamellar cataract).

The data presented in Fig. 2 clearly indicates that compared with the WT lens (Fig. 2*B*), the mutant lens is less heterogeneous (Fig. 2*A*). A notable feature of the high transcriptional heterogeneity in the single fiber cells isolated from of the developing postnatal mouse lens is the presence of a population of fiber cells within the cortical region, which show low crystallin and high noncrystallin gene expression. Although there are other groups of fiber cells which show specific gene activities in other regions of the lens, this group of fiber cells is an easily noticeable feature that becomes a molecular signature of heterogeneity in the WT lens (Fig. 2*B*) (7). It will be important to understand the regulation of gene activities in this group of cells. For instance, these cells show appreciable expression of *Hsf4* (7).

Although the status and functional role of these cells in the lens development remains speculative, their absence in the transgenic (cataract) lens (Fig. 2, *A–C*) suggests that they may have an important role in the progression of differentiation and, therefore, a role in generating the transparent phenotype of the lens. We surmise that *Hsf4* is involved, either directly or indirectly, in the emergence of these fiber cells. This also brings us to consider how *Hsf4* may be involved in generating the phenotype of low crystallin expression and high noncrystallin expression. Based on previously published work that suggests repressor as well as activator functions for Hsf4 (26, 27), a simple explanation would be that Hsf4 is a repressor of crystallin gene expression (and therefore, we see the presence of low crystallin expressing fiber cells in the WT lens). However, we also need to explain high noncrystallin gene expression in these cells (7). The thesis that Hsf4 may be an activator for all the noncrystallin gene activity seems unlikely.

The status of *Hsf4* in the developing ocular lens needs further elucidation. We initially demonstrated that Hsf4 interacts with the heat-shock promoter of the αB-crystallin gene (Cryab) (28). It is interesting that different mutations in *Hsf4* DNA-binding domain result in differential binding affinities for the various arrangements of the heat-shock element in the heat-shock promoter (29), suggesting differential gene activities with genes containing varied arrangements of the heat-shock promoter elements. This complicates the mechanistic regulation of crystallins *versus* the noncrystallins and of those genes that contain a canonical heat-shock promoter and those that do not (28, 30).

Assays of differential gene activity in the *Hsf4*-null lens have yielded varied results (21, 31, 32), including reduced γS-crystallin, reduced Bfsp 1 and 2 (cytoskeleton proteins) and loss of posttranslational modifications of αA-crystallin (32), and down-regulation of vimentin (33). Up-regulation of fibroblast growth factors (21), reported in *Hsf4*-null lens, could alter gene activities of noncrystallins as well as crystallins and many downstream genes including those involved in DNA damage stimulus (34). Any of these activities in an appropriate context, could contribute to the pathology. However, it is difficult to focus on relevant downstream gene activities in light of the fact that *Hsf4* knockout phenotypes are unlike the lamellar cataract phenotype, which is spatially and temporally restricted. An interesting report suggests involvement of Hsf4 through apoptosis regulators including p53 and its downstream genes in the zebrafish lens fiber cell differentiation (35).

Our previous work with different cell-types in culture (epithelial cells and fibroblasts) suggests that occupation of the heat-shock promoters of Cryab and Hsp70 (heat-shock protein 70) by Hsf1 and Hsf4 (as determined by ChIP) depends on cell-type–dictated access to each of these promoters (36).

At this time, we do not have a complete grasp of the presence and the extent of various cell-types in the developing fiber mass of the lens; yet, the data presented here do indeed put *Hsf4* at a critical place in the molecular progression that generates heterogeneity in the fiber cells of the cortical region. The absence of the group of cortical fiber cells (which show low crystallin expression and high noncrystallin expression) (Fig. 2, *A–C*) may represent a cell-type that is part of the molecular progression toward terminal differentiation. It is, however, difficult to align events temporally to pinpoint the involvement of Hsf4. It remains to be determined whether Hsf4 is directly involved in generating heterogeneity.

It is notable that the knockout of the *Hsf4* gene does not affect the early development of the lens but affects the postnatal development (21, 31, 32). The postnatal development of the ocular lens depends on the differentiation of fiber cells from the anterior epithelium at the lens equator (Fig. 1). The data obtained here with the postnatal day 2 of the developing lens points to a developmental “arrest.” Developmental arrest may lead to repetitive gene activity if there is a block in the molecular progression for which Hsf4 is required. This is supported by the data presented in Fig. 6, *E* and *G*.

The repetitive gene activity may lead to the absence of molecular transitions from one phase (region) to the other and, therefore, in the absence of transcriptional heterogeneity. Absence of transcriptional heterogeneity results in similar molecular composition in the fiber cells, in all the regions of the lens (namely the equatorial, the cortical, and the nuclear). This is reflected in the data presented in Fig. 4 where, compared with the WT, the cataract lens fiber cells from different regions show very little differential gene activity. Absence of differential gene activity also shows up as low variability in gene expression (Fig. 5, *B* and *D*). Because terminal differentiation is a progression through a number of cell-states, both morphological as well as molecular, our data do indeed suggest that heterogeneity is important by its very absence in the lamellar cataract paradigm.

Developmentally and morphologically, cortical fiber cells signify an intermediate state between the nascent fiber cells on the surface of the lens and the terminally differentiated fiber cells in the center or the nucleus of the lens. These fiber cells represent a molecular transition that connects nascent to terminal differentiation in the WT lens. Heterogeneity in these cells connotes the large variability in the expression of crystallin and noncrystallin gene activities.

It is obvious that although the cortical fiber cells in the WT and the mutant may be spatially comparable, at the molecular level, however, they may not be the same cells. The interruption in the developmental progression may have stopped the appearance of the cell-type(s), normally seen in the WT cortex.

As indicated above, 29% of all cortical fiber cells in the WT lens show poor expression of crystallins coupled with high expression of noncrystallin genes (7). This group of cells contributes >80% of variance to the gene expression data in the WT lens. This is particularly significant, because the equatorial and the nuclear fiber cells show comparatively low variability (Fig. 5). Thus, the question arises: Is this heterogeneity consequential? In other words, does transcriptional heterogeneity at the single cell level have a role in the final realization of the functional phenotype of this tissue? The functional phenotype translates into the coordinated expression of specific crystallins in the nuclear fiber cells, which make the visual axis (7). This does not happen in the transgenic (cataract) lens (Fig. 8).

It is tempting to speculate that transcriptional heterogeneity may assign molecular identity to each fiber cell or a group of fiber cells, which may be crucial for progression to terminal differentiation. We submit that without this heterogeneity, the molecular progression stalls. The repetitive expression patterns seen in the transgenic mutant lens (Fig. 6, *E* and *G*) may simply be the result of repetitive “searching” for these identities. Lack of heterogeneity (Figs. 2–5) and the ensuing repetitive gene expression (Fig. 6) could then manifest as susceptibility to the eventual disease phenotype, the cataract pathology (Fig. 1*B*).

It is interesting that the expression of crystallins in the transgenic (cataract) lens fiber cells is not lost or down-regulated but misplaced (Fig. 8). This suggests that the molecular progression of terminal differentiation has stalled, whereas the repetitive pattern manifests as misplaced expression. We have represented this schematically in Fig. 8, *C* and *D*.

In the transgenic (cataract) lens, because crystallins are expressed all over the fiber cells in different regions, we can deduce that the crystallin expression *per se* does not provide the spatial identity to the fiber cells; those identities, we surmise, must come from other gene activities/cells. Alternatively, functional differentiation may depend on the spatial and/or morphological accuracy of the gene activity (for example of crystallins) without which specific molecular identities/cell-types will not emerge. These molecular identities must conform with the spatial identities. In the lens this is critical because the shape and the location of the fiber cell dictates how the light is refracted (3, 37, 38).

The expression of nine crystallins, namely Cryaa, Crybb3, Cryba1, Crygf, Crygb, Cryge, Crybb1, Cryga, and Crygc, is coordinately regulated right from when the cells enter differentiation at the equator, up to the point when they enter terminal differentiation in the lens nucleus (7). Fig. 7*A* shows the coordinated expression of these nine crystallin genes. What is notable, however, is that although the cortical gene activity shows a downward trend, the expression of these nine genes keeps going up as the terminal differentiation sets in (in the nuclear fiber cells) (7). Quite significantly, the fiber cells in the cataract paradigm present a very different (uncoordinated) expression of these genes (Fig. 7*B*). Curiously, Cryaa, a gene for the predominant structural protein of the ocular lens whose expression along with the rest of the crystallins is dysregulated, has been reported to be associated with lamellar cataract phenotype (39).

Finally, it is important to recap that the transgenic paradigm studied here was generated by the disruption of the *Hsf4* gene activities (19, 20). The data presented in this investigation suggest that disruption of the Hsf4 activity disrupts cellular progression of development, concurrent with the impairment of the variability of gene expression in the PND02 lens. This is highly significant because the absence of heterogeneity seems to accompany the derangement of the developmental program, which finally proves pathological (appearance of a cataract).

One visible phenotype of the *Hsf4* mutation is the persistence of nuclei related to the absence of DNase2β activity known to be associated with nuclear cataracts (40). It is possible that the homogeneity of the gene expression (which accompanies the pathological retention of nuclei in the differentiating fiber cells) (Fig. 1*B*, cataract PND02) represents the molecular derangement that foreshadows the morphological appearance of the pathology at PND25 (Fig. 1*A*). This possibility, however, must await additional work to chart out the molecular progression between the PND02 and the appearance of cataractogenesis at PND25 (Fig. 1*A*).

It is important to point out that in the lamellar cataract, initially, only a few fiber cells become opaque/cataractous (Fig. 1*A*). This suggests heterogeneity in the molecular vulnerability of fiber cells to the disruption of *Hsf4*. Transcriptional heterogeneity thus clearly contributes to differential vulnerability to pathogenesis. The paradigm delineated here should allow us to seek a cellular basis for the requirement for heterogeneity, which must involve identification (and functional characterization) of various cell types within the three-dimensional space of this tissue.

## Experimental procedures

### Animals

C57Blk/6Ncrl mice were purchased from Charles River Laboratories International. Embryonic day 14 (E14) pregnant mothers were acclimatized for a week with appropriate diet and water *ad libitum*. Transgenic mice were generated and maintained in the Department of Laboratory and Animal Medicine (DLAM) facilities at the University of California, Los Angeles, CA (UCLA) (19). All the work with animals followed the institutional guidelines and protocols approved by Animal Research Committee (UCLA). Postnatal day 2 (PND02) mice pups were euthanized, the eyes were enucleated and soaked briefly in mammalian Ringer's solution (150 mm NaCl, 5.4 mm KCl, 2 mm MgCl_2_, 2 mm CaCl_2_, in 20 mm Hepes, pH 7.4, 300 to 310 mOsm). The lenses were dissected and stained (19).

### Isolation of single fiber cells

The single fiber cells from the PND02 transgenic lens were isolated based on a previously published procedure for adult lenses (41). This procedure was modified for isolation of fiber cells from a developing postnatal day 2 (PND02) mouse lens (7).

Our procedure is based on the temporal release of fiber cells from a PND02 mouse lens by gentle shaking in a buffer. The lenses with capsules intact (∼1000 μm in diameter, weighing about 10–12 mg) were isolated from enucleated eyes and rinsed with mammalian Ringer's buffer. The lens was incubated for 10 min at 33 °C in SHE solution (280 mm sucrose, 10 mm Hepes, pH 7.4, 10 mm Na-EDTA, 300 to 310 mOsm) containing 0.5 mg/ml trypsin (Life Technologies). The temperature was increased gradually (1 °C/min) to 37 °C, and the incubation continued for next 5 min. After this incubation, the lens was rinsed four times with SHE solution (now without trypsin), and the epithelium and capsule gently removed by making a small incision at the equator.

The intact fiber mass was rinsed with SHE solution (without trypsin) and incubated in the same solution for 5 mins at room temperature. It was then transferred to a tabletop shaker (circular rotation, one revolution per second). The superficial fiber cells start coming off from the fiber mass. At the end of 10 min, the SHE solution containing fiber cells was smeared on a Petri dish from where the cells were collected individually by aspiration using a 10 μl pipette tip. Fibers collected in the first 10 min were labeled as equatorial fibers (length, 100 to 120 μm). The rest of the fiber mass was rinsed twice with SHE solution and transferred to a new well. The shaking was continued for the next 10 min and the fiber cells were collected as before. These were labeled as cortical fibers (length, 220 to 240 μm). The remaining fiber mass was gently rinsed once with the SHE solution and a coverslip was placed on top of the fiber mass and the fibers were dissociated with gentle tapping. The coverslip was removed and the loose suspension transferred to a fresh slide from where the nuclear region fibers (length, 600 to 640 μm) were collected, one at a time. In all, we collected 92–96 single fiber cells from one lens (*n* = 30–32 single fiber cells from each region) in about 45 min. Only one lens was processed at one time. In this manual procedure, a low percentage of cells may belong to the transition between two contiguous regions and, therefore, be counted as part of one of the two alternative regions, namely equatorial or cortical and cortical or nuclear. The isolation procedure was kept as short as possible to avoid morphological or physiological changes. The viability of the isolated fiber cells was ascertained by a trypan blue exclusion assay. The whole procedure takes about 45 min; the cells retain their morphological and molecular integrity (7).

### Assessment of gene expression by quantitative PCR (qPCR)

The single-step RNAzol RT (MRC, Inc. Cincinnati, OH) method was used for RNA isolations from single fiber cells. cDNA synthesis and the qPCR were done using the Biomark Microfluidic System (Fluidigm Inc., Palo Alto, CA). A detailed systematic procedure is described in Gangalum *et al.* (7). RNA from individual fiber cells was interrogated with probes for 95 genes (Table S1).

### Data analysis

In this investigation, we present analysis of data generated from five different lenses using five Biomark chips. At one time, the Biomark Microfluidics System processes only one chip (96 samples). Each chip accommodates fiber cells isolated from one lens (30–32 fiber cells from each morphological zone of differentiation). In one chip we interrogate 92 single fiber cell RNAs (isolated from one lens), one reaction for no template control and three dilutions of total lens fiber mass RNA.

We present here a cumulative merge of the data obtained from five Biomark IFC (integrated fluidic circuits) chips (5 lenses × 96 samples = 480 samples/fiber cells). The raw data generated from Biomark (Fluidigm Inc.) in .csv format was analyzed using SINGuLAR software in the R or R studio. To start the analysis, we need the following three files: 1) Data file as .csv file (the gene expression data file), 2) sample list in .txt (equatorial, cortical, and nuclear fiber cells, total *n* = 460 single fiber cells), and 3) gene list in .txt format (should contain two gene groups: Crystallins (*n* = 17) and noncrystallins (*n* = 77); total = 94 genes) for the analysis in the WT fiber cells. We used the same list of genes with the addition of eGFP (enhanced GFP gene sequences) (total = 95) for analysis of the fiber cells isolated from the transgenic mutant lens.

The autoAnalysis() command runs the data and saves the data as object file known as exp(auto_analysis).fso file in a prespecified folder. This .fso file can be opened using fluidigmSCObjectToExcel file and then saved as .csv file or .xlsx file. The .xlsx file contains raw Ct values, Expression (log2) values, type of analysis performed (ANOVA, Pearson R correlation), number of samples, number of genes, and a summary of data analysis in individual sheets. After removing outlier information (such as samples with expression values above 24 Cts or limit of detection (LoD) or samples that show no amplification), the linear expression values were calculated using this formula = 2^(LOD Ct − Measured Ct)^ or 2̂^log2expression^. For example, if the LoD is a Ct of 24, then the Expression (log2) of 24 minus measured Ct value is calculated.

The singular software (Fluidigm_SC 3.6.2 version) was used to analyze and generate the heat clusters, violin plots, box plots, and PCA and tSNE analysis. We have also used custom “R” programs and other packages compatible in “R” (corrplot, plotly, ggplot2, calibrate & reshape2) or python (version 3.7.1) package (seaborn 0.90) to generate heat-maps, violin plots, correlation matrix plots, volcano plots, and box plots presented in the manuscript.

Unsupervised hierarchical heat map clusters in Fig. 2 and Figs. S1–S3 were represented in global *Z* scores (*blue* = low expression, *red* = high expression). The global *Z* scores were calculated using this formula, *Z* = (*x* − μ)/(σ/√*n*), where *x* = global mean of all single cells, μ = sample mean of all genes in a single cell, σ = S.D., *n* = total number of single cells. The clustered heat map of global *Z* score display is based on the sample similarity, which uses normalized expression values with global mean and global S.D.

PCA (plots in Fig. 3*A*) or *t*-distributed stochastic neighbor embedding analysis (tSNE in Fig. 3*B*) are the two types of data reduction methods that allow multidimensional, single-cell data sets generated from multiplex qPCR for the plotting purposes and visual variance analysis. The objective of running PCA is to interpret the variation within a data set with as many variables as possible while retaining the large variation information. The PCA reduces the dimensionality of a data set by transforming it into a new set of uncorrelated variables, called principal components (PCs), with decreasing degrees of variability. The first two to three PCs (PC1, PC2, and PC3) explain most of the variation in the data set. Each successive PC in turn explains the next highest variance for the data, under the constraint that its relationship with the previous PC is zero. The PCA algorithm in the SINGuLAR Analysis Toolset uses successive orthogonal transformations (with defined lengths and angels of the vectors) to convert data into a series of PCs that explain variance in the data (Fluidigm Inc.).

Similarly, the tSNE is a nonlinear, probabilistic dimensionality reduction method that takes a set of data points in a high-dimensional space and finds faithful representation of those points in a lower-dimensional space. The lower-dimensional space is typically a two-dimensional scatter plot (tSNE1 and tSNE2) with apparent clusters at several scales. In such a scatter plot, tSNE can capture much of the local pattern of the high dimensionality data while also revealing its global pattern.

Correlation analysis shown in this manuscript (Fig. 7) is the linear association between two variables. Values of the correlation coefficient always lie between −1 and +1 (as Pearson R value), with +1 indicating that two variables are positively correlated and 0 indicating no correlation or linear relationship, whereas −1 indicates negative correlation. The correlation coefficient measures only the degree of linear association between two variables and not the causality. The correlation coefficients were calculated by the Pearson method. We used SINGuLAR Analysis Toolset User Guide, Biomark, Fluidigm Inc. for all data analysis and generation of figures presented in this manuscript.

For analysis presented in Fig. S1, the data obtained from Biomark was imputed with a Ct value cutoff of 24 for samples, which were below detection limit, as per the manufacturer's instructions. Gene-specific *Z* scores were visualized using a heat map. Complete linkage hierarchical clustering method (Fig. S1) was used to formally test for the region effects. Linear mixed effect models were employed for fixed region effects (cortical and equatorial *versus* nuclear), fixed mouse effects (for the five mice), and for a random mouse-region effect to assess correlations between samples from the same region of the same mouse. The estimates, the 95% confidence intervals, and the *p* values of the region effects for each gene were determined. In addition, we used adjusted Type 3 test *p* values for comparing the expression levels across all regions. We corrected for multiple testing using the Benjamini-Hochberg procedure (42); here we report the FDR-adjusted *p* values. The FDR threshold was set at 5%. R software (R version 3.4.1) was used for the analysis (Fig. S1).

Note: All the WT data in this manuscript that were used for comparative analyses were obtained from Ref. 7.

## Author contributions

S. P. B. and R. K. G. conceptualization; S. P. B. resources; S. P. B., R. K. G., D. K., and S. M. data curation; S. P. B., R. K. G., D. K., R. K. K., X. Z., and D. E. software; S. P. B., R. K. G., D. K., S. M., X. Z., and D. E. formal analysis; S. P. B., R. K. G., and D. E. supervision; S. P. B. funding acquisition; S. P. B. and R. K. G. validation; S. P. B. and R. K. G. investigation; S. P. B., R. K. G., and R. K. K. visualization; S. P. B. and R. K. G. methodology; S. P. B. and R. K. G. writing-original draft; S. P. B. project administration; S. P. B. and R. K. G. writing-review and editing.

## Supplementary Material

Supporting Information

## Footnotes

2 The abbreviations used are:

PND: postnatal day
PCA: principal component analysis
qPCR: quantitative PCR
eGFP: enhanced GFP
PC: principal component
tSNE: *t*-distributed stochastic neighbor embedding analysis
Ct: threshold cycle
FDR: false discovery rate
ANOVA: analysis of variance.
