# Supplementary material for: Transcriptional profiling of single fiber cells in a transgenic paradigm of an inherited childhood cataract reveals absence of molecular heterogeneity
Source: J Biol Chem. 2019 Jun 26;294(37):13530–44. doi: 10.1074/jbc.RA119.008853 (PMC6746439; doi:10.1074/jbc.RA119.008853)
Supplement: Supporting Information [file supp_294_37_13530__index.html]

Transcriptional profiling of single fiber cells in a transgenic paradigm of an inherited childhood cataract reveals absence of molecular heterogeneity — Cataract lens fiber cells lack transcriptional heterogeneity — Transcriptional profiling of single fiber cells in a transgenic paradigm of an inherited childhood cataract reveals absence of molecular heterogeneity — Cataract lens fiber cells lack transcriptional heterogeneity — Supporting Information 

# Transcriptional profiling of single fiber cells in a transgenic paradigm of an inherited childhood cataract reveals absence of molecular heterogeneity

## Supporting Information

- Supporting InformationJBCRevised05232019 - Revised Supporting Information
