# Supplementary material for: Transcriptional profiling of single fiber cells in a transgenic paradigm of an inherited childhood cataract reveals absence of molecular heterogeneity
Source: J Biol Chem. 2019 Jun 26;294(37):13530–44. doi: 10.1074/jbc.RA119.008853 (PMC6746439; doi:10.1074/jbc.RA119.008853)
Supplement: Supporting Information [file supp_RA119.008853_144875_2_supp_351361_ptfrcr.pdf]

Supporting Information

Transcriptional Profiling of Single Fiber Cells in a Transgenic Paradigm of an Inherited Childhood Cataract Reveals Absence of Molecular Heterogeneity

Suraj P Bhat[1, 2,3,*] Rajendra K Gangalum[1], Dongjae Kim[1], Serghei Mangul[4], Raj K. Kashyap[1], Xinkai Zhou[5], David Elashoff[5]

[1]*Stein Eye Institute, Geffen School of Medicine,* [2]*Brain Research Institute,* [3]*Molecular Biology Institute,* [4]*Department of Computer Science and Human Genetics,* [5]*Department of Medicine, University of California, Los Angeles, CA 90095-7000*

Running Title: Cataract lens fiber cells lack Transcriptional heterogeneity

Includes:

Figs. S1 to S6 and Table S1

Supplemental Table S1: Title: A list of the genes and gene sequences used for the interrogation of single fiber cells in this study.

Figure S1

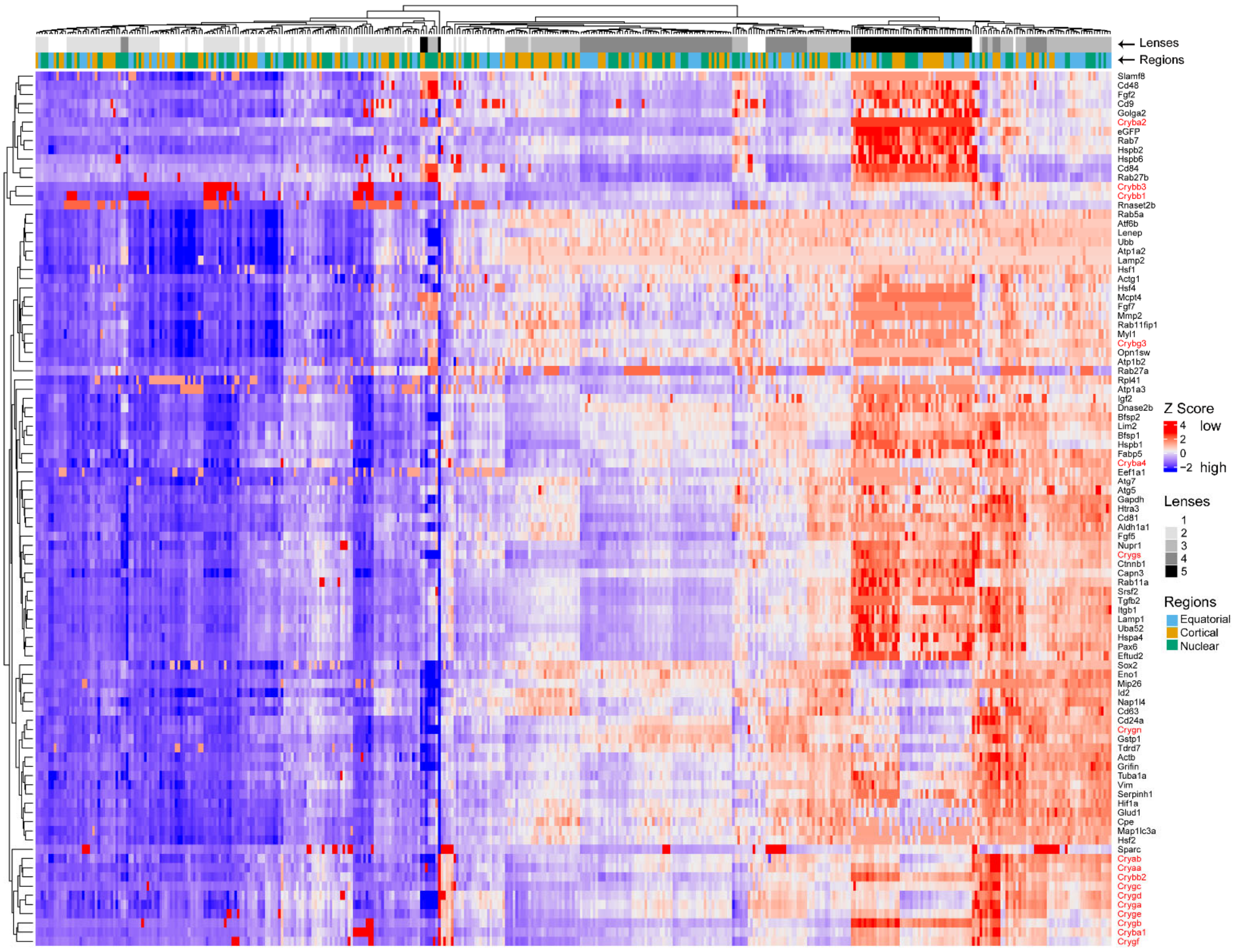

Figure S1. Complete linkage hierarchical clustering of gene expression in single fiber cells of the mutant (Cataract) lens.

The Ct value for each gene is converted to a Z-score (red – low expression and blue-high expression) and represented in the heat map. Although lens (animal) is more discernible here, by using linear mixed effect model, we found that the gene expression in single fiber cells isolated from different regions (Equatorial, Cortical and Nuclear) from each lens correlates well with 95% confidence intervals.

Figure S2

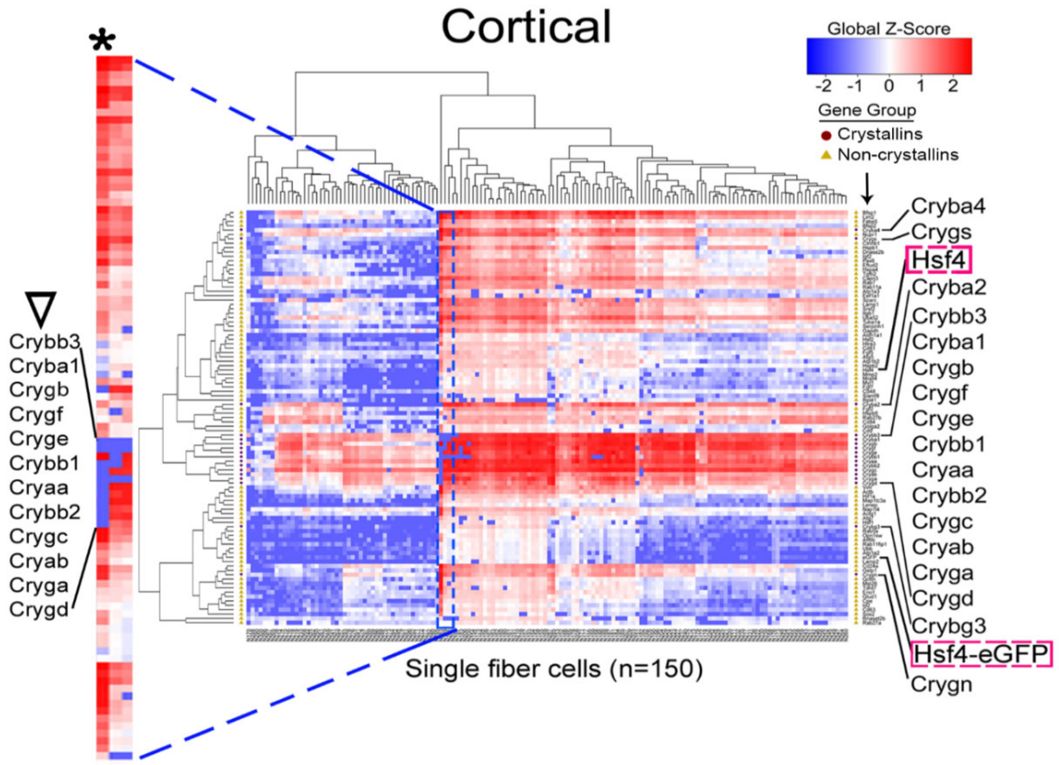

Figure S2. Fig S2. Heat map of gene expression in cortical fiber cells isolated from a transgenic cataractous lens

Heat map of gene expression (z-score) of single cortical fiber cells (n=150) isolated from five transgenic lenses. In the cumulative analysis of 460 fiber cells presented in Fig. 2C, we identified a fiber cell) with significantly low expression of crystallin and high expression of non-crystallin genes. Here we identify this fiber cell to be of cortical origin (magnified, left). Gene groups are indicated on y-axis (right) and the number of fiber cells on the x-axis. This fiber cell is sample # N283 in Fig. 2C. Heat maps generated for equatorial and nuclear fiber cells are not shown.

Figure S3 (A, B, C)

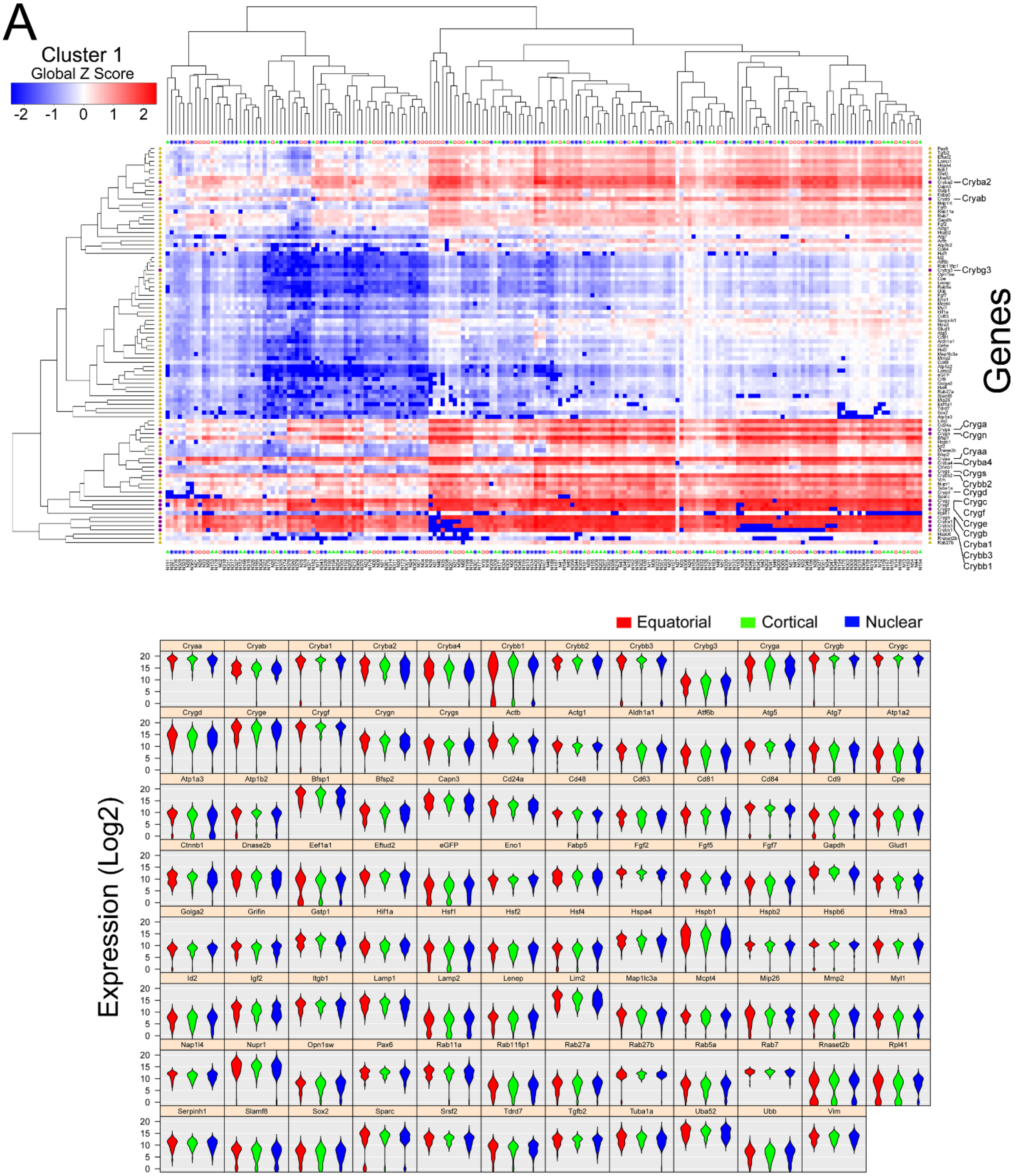

Figure S3

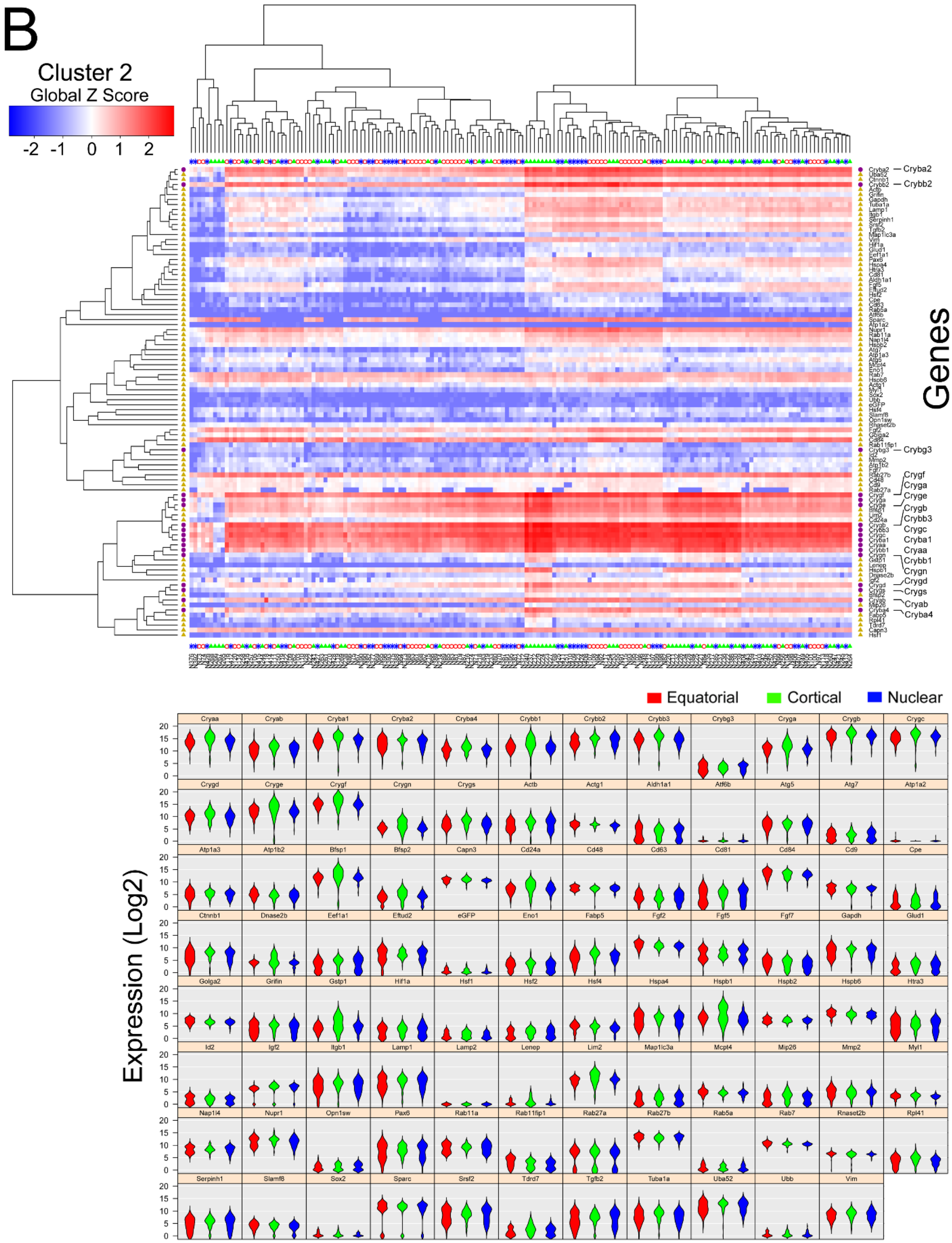
B
Cluster 2
Global Z Score
-2 -1 0 1 2
Genes
Cryba2
Crybb2
Crybg3
Crygf
Cryga
Cryge
Crygb
Crybb3
Crygc
Cryba1
Cryaa
Crybb1
Crygn
Crygd
Crygs
Cryab
Cryba4
Equatorial
Cortical
Nuclear
Expression (Log2)

Figure S3

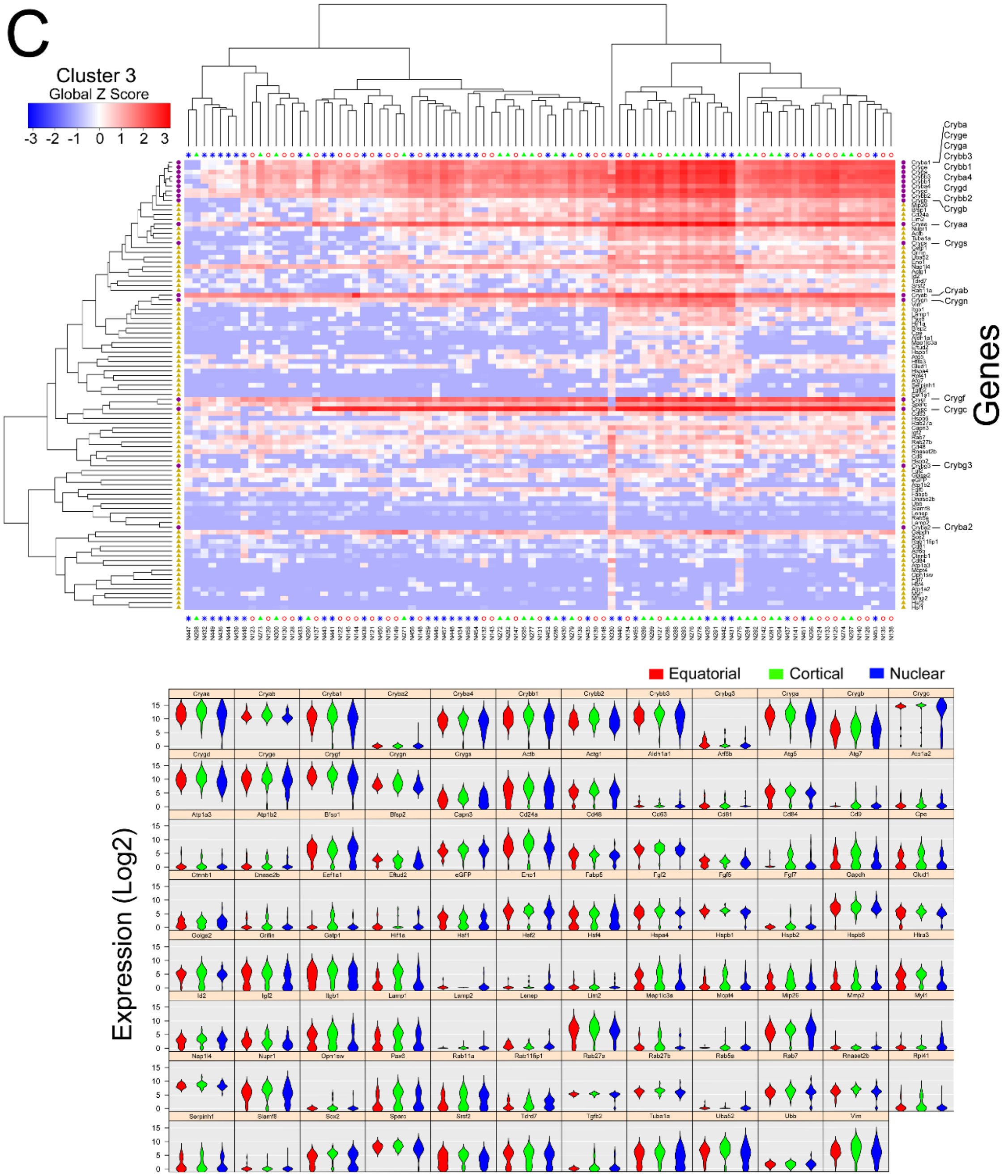
C
Cluster 3
Global Z Score
-3 -2 -1 0 1 2 3
Cryba
Cryge
Cryga
Crybb3
Crybb1
Cryba4
Crygd
Crybb2
Crygb
Cryaa
Crygs
Cryab
Crygn
Crygf
Crygc
Crybg3
Cryba2
Genes
Equatorial
Cortical
Nuclear
Expression (Log2)

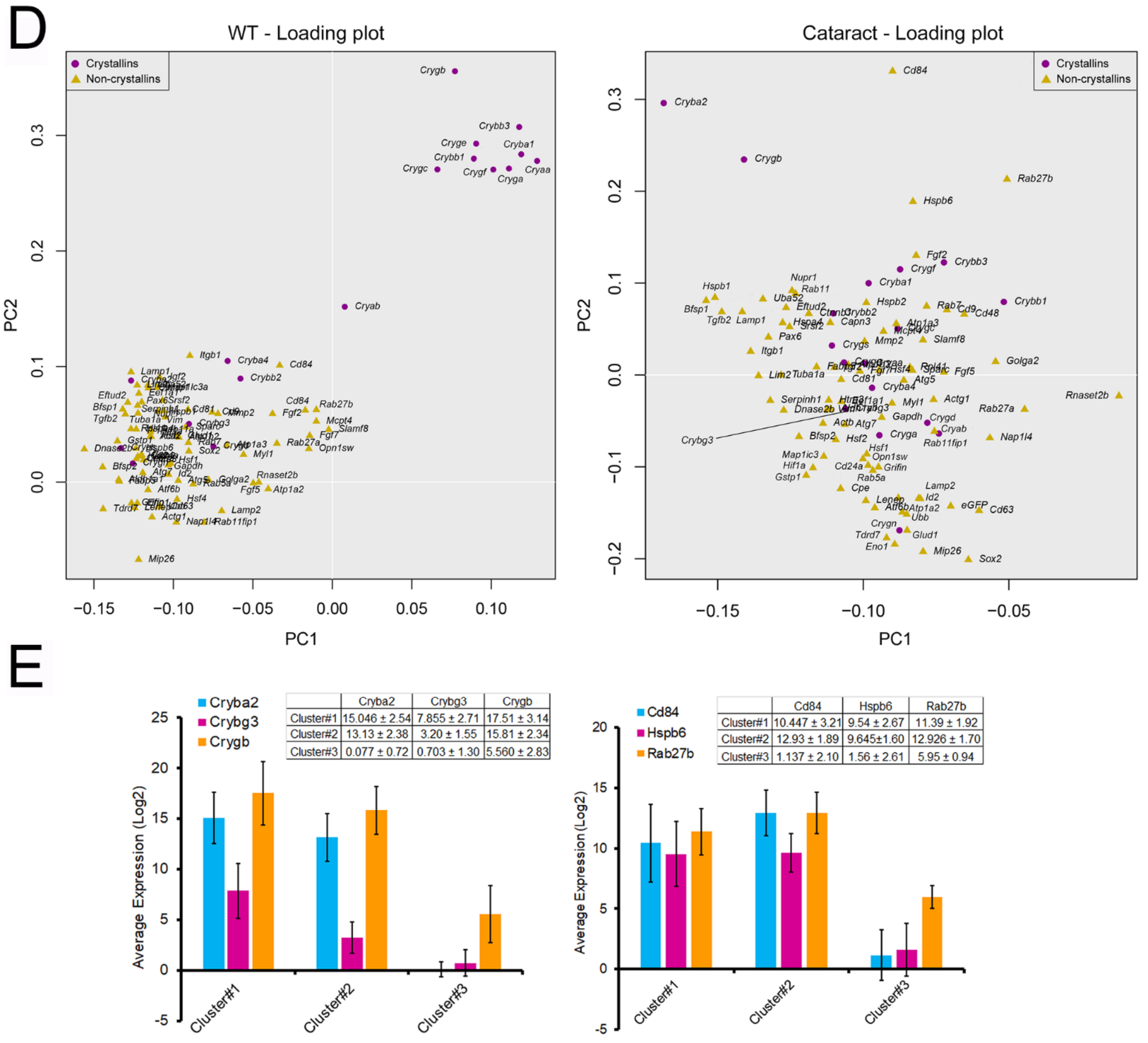

	Cryba2	Crybg3	Crygb
Cluster#1	15.046 ± 2.54	7.855 ± 2.71	17.51 ± 3.14
Cluster#2	13.13 ± 2.38	3.20 ± 1.55	15.81 ± 2.34
Cluster#3	0.077 ± 0.72	0.703 ± 1.30	5.560 ± 2.83

	Cd84	Hspb6	Rab27b
Cluster#1	10.447 ± 3.21	9.54 ± 2.67	11.39 ± 1.92
Cluster#2	12.93 ± 1.89	9.645±1.60	12.926 ± 1.70
Cluster#3	1.137 ± 2.10	1.56 ± 2.61	5.95 ± 0.94

Figure S3. Detailed analysis of the three clusters produced by PCA in Fig. 3

A. Cluster #1 (n=187)**, e**quatorial (red, n=58), cortical (green, n=58) and nuclear (blue, n=71). Heat map of gene expression (z-scores) (top) and violin plots (bottom) of single fiber cells is presented. All 16 crystallins, except Crybg3 are expressed at high levels. Note low expression of non-crystallins (~50 genes) in almost all fiber cells in this cluster. However, there are 30 non-crystallin genes that are expressed in >70% of the fiber cells. Lamp1, Uba52, Capn3, Lim2, Bfsp1, Hspb1, Itgb1 and Nupr1 show robust expression in majority of the fiber cells; their violin plots (bottom) display remarkable similarity in their distributions across fiber cells, indicating less variation in the population of fiber cells. **B. Cluster #2** (n=168), equatorial (red, n=58), cortical (green, n=60) and nuclear (blue, n=50). Again, there is high expression of all 16 crystallins except Crybg3. A large number of genes (~50 genes) show low expression.

Uba52, Fgf2, Nupr1, Cd84, Capn3, Lim2, Bfsp1, Rab7 and Hspb6 are appreciably expressed genes in majority of the fiber cells. Relative expression levels of crystallins are high compared to non-crystallins in this cluster. **C. Cluster #3** (n=89)**,** equatorial (red, n=30), cortical (green, n=27) and nuclear (blue, n=32). The fiber cells here show high expression of 14 crystallin genes except Cryba2, Crybg3 and Crygs. About 40 genes show low expression (low z-scores) in almost all fiber cells. Cd24a, Vim, Mip26, Bfsp1, Lim2, Nupr1, Actb, Gstp1, Grifin are the moderately expressed genes in majority of the fiber cells. **D. PCA loading plots** of WT (n=94 genes) and Cataract (n=95 genes) identify clusters of genes based on first two principle components, which show maximum variance. In the WT loading plot, nine cystallin gene (Cryaa, Cryba1, Crybb3, Cryga, Crygf, Cryge, Crybb1, Crygb and Crygc) cluster is clearly identified. Although a few gene names may not be visible because of overlap, the useful information is not compromised. This cluster is missing in the cataract. Most of the genes in the single fiber cells in the cataract lens show low variance. We have tabulated in E, three crystallins (Cryba2, Crygb and Crybg3) and three non-crystallin genes (Cd84, Hspb6 and Rba27b) that show differential presence in each cluster. **E.** Bar graphs show expression (log2) (Mean±SD) for three clusters (Cluster#1 (n=187); Cluster#2 (n=168) and Cluster#3 (n=89)) represented by w three crystallins (Cryba2, Crygb and Crybg3) and three non-crytallin genes (Cd84, Hspb6 and Rba27b). The Mean±SD values are tabulated (see Inset).

Figure S4

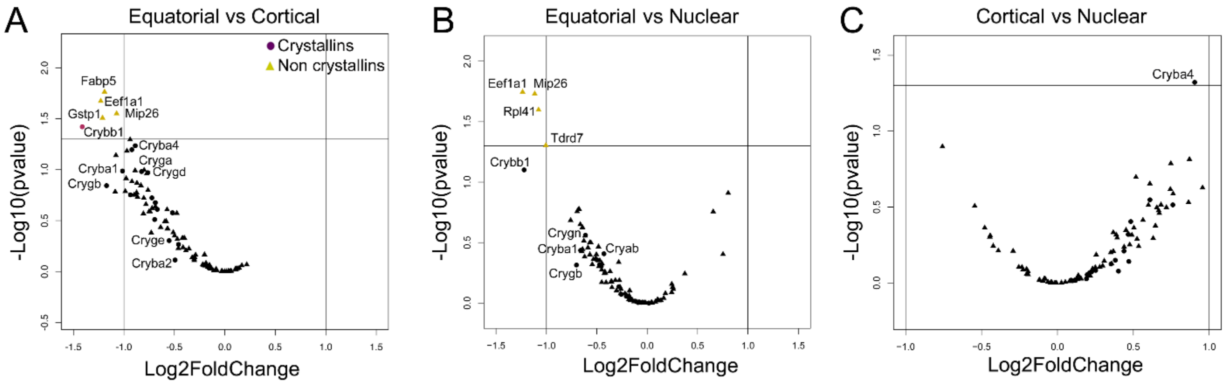

Figure S4. Absence of differentially expressed of genes in fiber cells isolated from equatorial, cortical and nuclear regions of the transgenic lens.

Volcano plot for differentially expressed genes in three comparisons (equatorial vs cortical, equatorial vs nuclear and cortical vs nuclear fiber cells). X-axis = log2fold change, y-axis = -log10 p value. Crystallins = ●, Non-crystallins = ▲. **A**. Equatorial vs cortical region show 5 (Fabp5, Eef1a1, Gstp1, Mip26 and Crybb1) differentially expressed genes ($p<0.05$). **B**. The equatorial vs nuclear region show four differentially expressed genes (Eef1a1, Mip26, Rpl41 and Tdrd7) ($p<0.05$). **C**. Cortical vs nuclear comparison shows merely one differentially expressed gene, Cryba4.

Figure S5 (A, B, C)

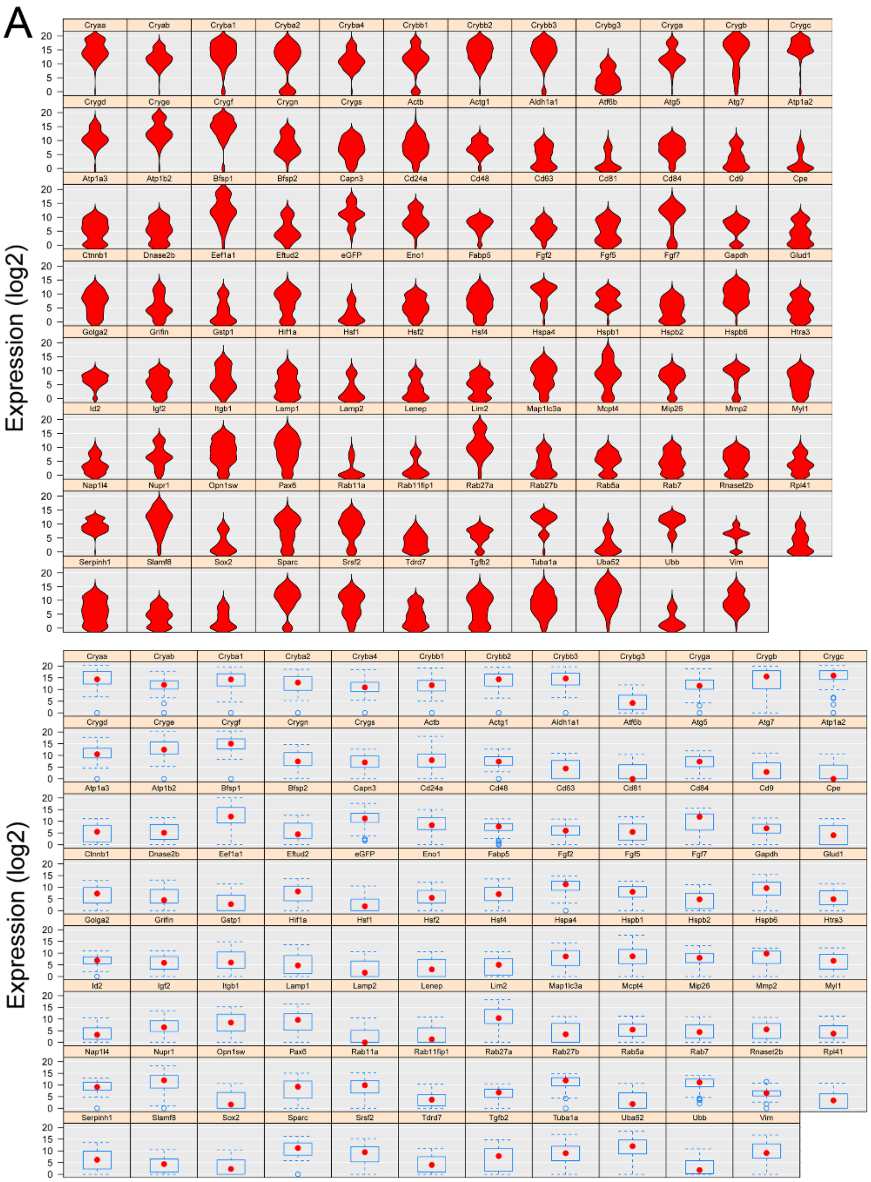

Figure S5

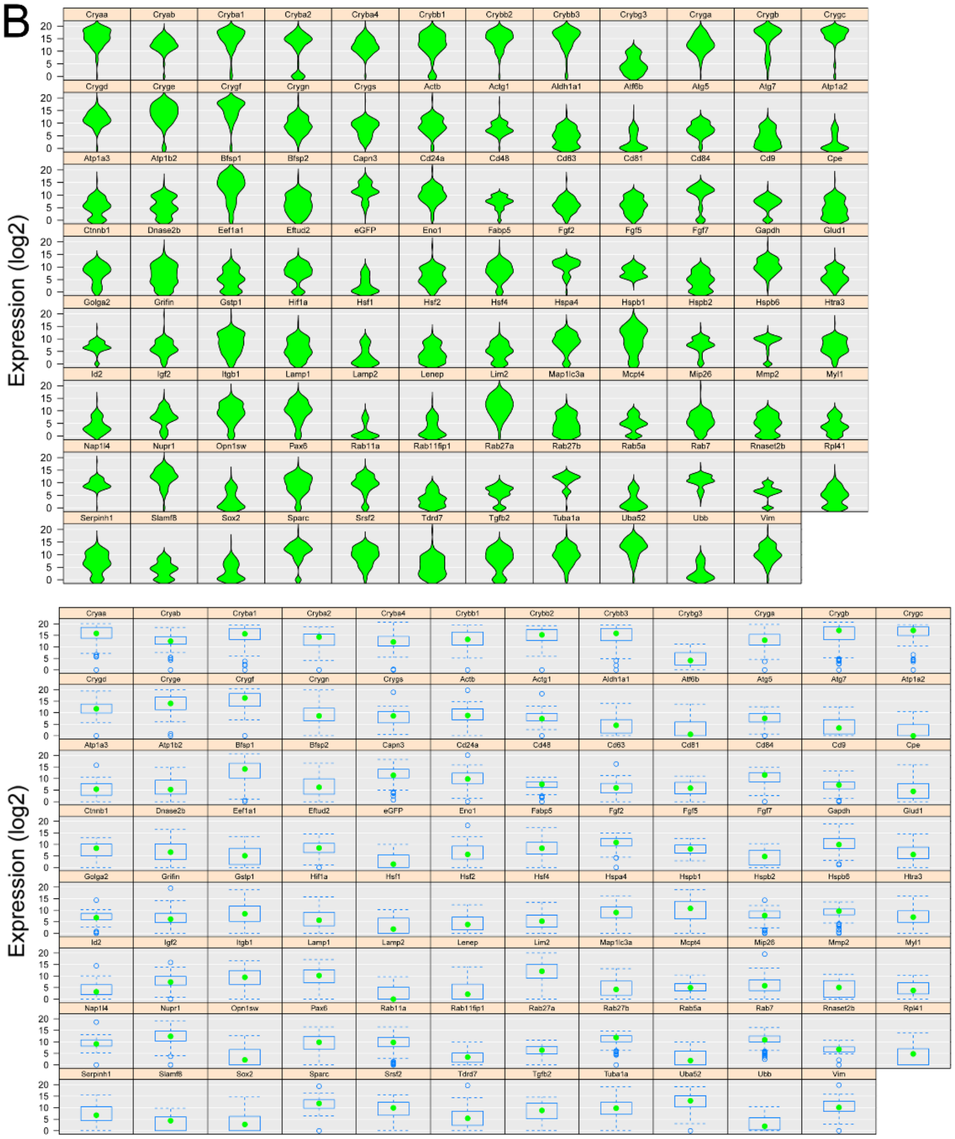
B
Expression (log2)
0 5 10 15 20
Cryaa Cryab Cryba1 Cryba2 Cryba4 Crybb1 Crybb2 Crybb3 Crybg3 Cryga Crygb Crygc
Crygd Cryge Crygf Crygn Crygs Actb Actg1 Aldh1a1 Atf6b Atg5 Atg7 Atp1a2
Atp1a3 Atp1b2 Bfsp1 Bfsp2 Capn3 Cd24a Cd48 Cd63 Cd81 Cd84 Cd9 Cpe
Ctnnb1 Dnase2b Eef1a1 Eftud2 eGFP Eno1 Fabp5 Fgf2 Fgf5 Fgf7 Gapdh Glud1
Golga2 Grifin Gstp1 Hif1a Hsf1 Hsf2 Hsf4 Hspa4 Hspb1 Hspb2 Hspb6 Htra3
Id2 Igf2 Itgb1 Lamp1 Lamp2 Lenep Lim2 Map1lc3a Mcpt4 Mip26 Mmp2 Myl1
Nap1l4 Nupr1 Opn1sw Pax6 Rab11a Rab11fip1 Rab27a Rab27b Rab5a Rab7 Rnaset2b Rpl41
Serpinh1 Slamf8 Sox2 Sparc Srsf2 Tdrd7 Tgfb2 Tuba1a Uba52 Ubb Vim

Figure S5

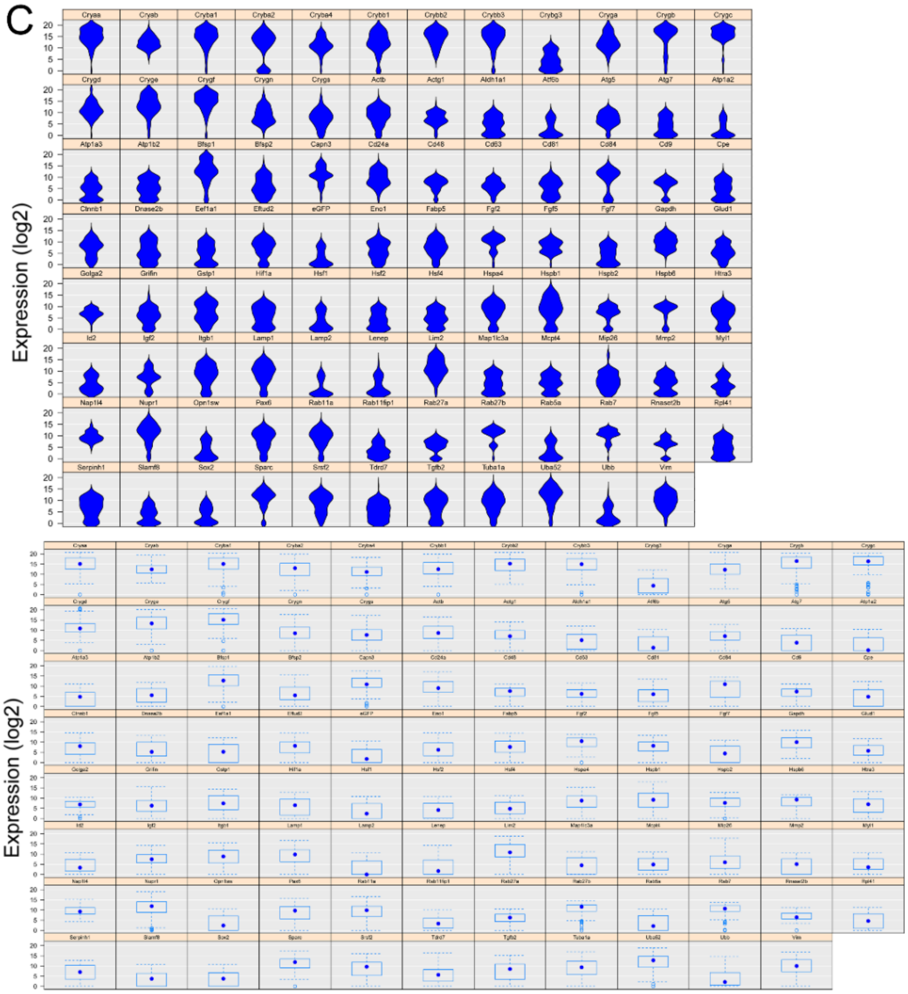

Figure S5. Complete violin and box plots of gene expression in fiber cells from different regions of the transgenic mutant lens.

A. Equatorial fiber cells. The violin plots of gene expression in equatorial fiber cells (red, top) and box plots of gene expression (red medians, bottom). Y-axis = expression (Log2), x-axis = probability of fiber cell distribution. In the equatorial fiber cells there is high expression of crystallins, except Crybg3. Box plots reveal narrow range of quartile distribution of gene expression in majority of the genes. **B. Cortical fiber cells.** The definitive multimodality seen in the WT cortical fiber cells is missing in this data. Box plots reveal significantly narrow range of quartile distributions in all genes studied in this data set. **C. Nuclear fiber cells.** Other than the similarity of expression distributions among crystallins in the three regions (for example the very first two genes Cryaa and Cryab), we also see a striking similarity of the patterns for the transcription of many genes in all regions (red, green and blue). Box plots reveal narrow range of quartile distribution of gene expression in most genes in all regions.

Figure S6

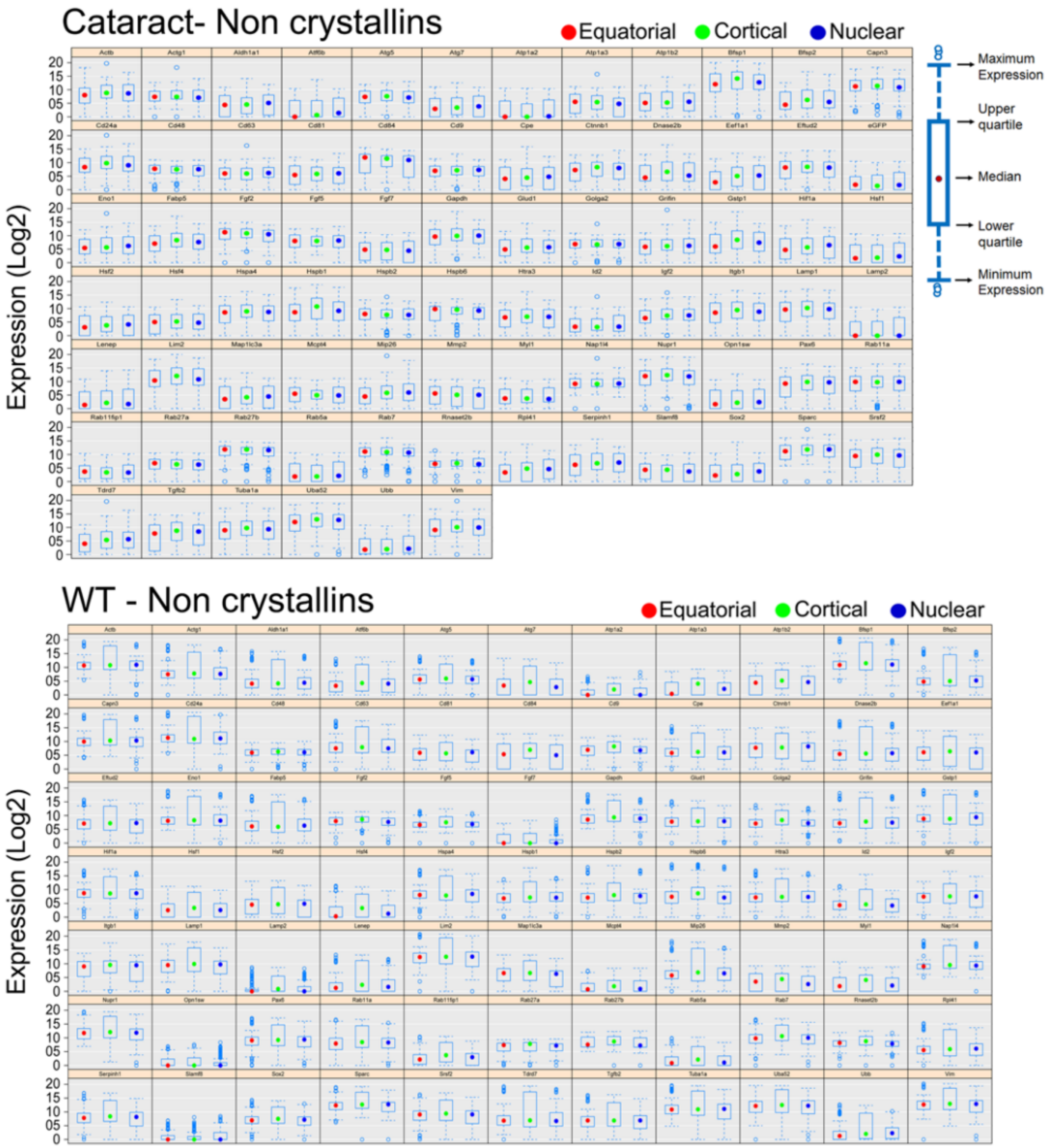

Figure S6. Box plots of non-crystallin gene expression in the fiber cells isolated from three different regions of cataract and wild type lenses.

Data from single fiber cells isolated from equatorial (green dot), cortical (red dot) and nuclear (blue dot) regions of wild type lenses (n=446) (# of genes =77) and transgenic cataract lenses (n=460) (#of genes =78) is presented. Y-axis =

expression (Log2), x-axis = number of fiber cells. The box plot (a schematic legend is shown on the top right) displays, median value (filled circle), with upper and lower quartile range (rectangular box) and minimum as well as maximum expression values (dotted lines). Box plots of crystallin gene expression are presented in Fig. 6. In the cataract fiber cells (top), significant number of genes show moderate to low (<10 expression (Log2)) expression profiles except Bfsp1, Lim2, Nupr1 and Uba52, which show >10 expression (Log2) values. Note the compactness of the boxes in the cataract fiber cells (indicating narrow ranges of expression) in comparison to that in the WT fiber cells (bottom). Overall, the expression profiles of fiber cells derived from equatorial, cortical and nuclear regions of cataract lenses show similar distribution profiles. On the other hand, many of the non-crystallin genes in the cortical fiber cells derived from the wild type lenses show moderate to high (>10 expression (Log2)) values, with wide range of distribution patterns with high variation.

Table S1. Gene list and primer sequences

This table contains the gene names with forward and reverse primer sequences used in the microfluidic qRT-PCR assays (Biomark HD, Fluidigm Inc.,) to generate the data from single lens fiber cells isolated from the WT and transgenic (cataract) lenses. (An Excel file with complete Gene Ontology and other details will be submitted if required).

Gene Symbol	Forward Primer	Reverse Primer
Actb	CCCTAAGGCCAACCGTGAAA	AGCCTGGATGGCTACGTACA
Actg1	GCTTACACTGCGCTTCTTG	GTGCGGCGATTTCTTCTTC
Aldh1a1	AGACCTGGATAAGGCCATCA	CACTGGGCTGACAACATCA
Atf6b	GTCGACGGAAGATACCTCACA	ACAGGTTTCACTGGAGGAGAC
Atg5	CAGCTCTTCCTTGGAACATCAC	GCATCCTTGGATGGACAGTGTA
Atg7	GCAGTGATGACCGCATGAA	TCGAACCGTGACAGAAAACC
Atp1a2	AGCTGGGCCGAAAATACCAA	TGGGTCCATCTCTAGCCAGAA
Atp1a3	CCCTCAGCAGAAGCTCATCA	ATTCACACCATCGCCAGTCA
Atp1b2	ACTTCTATGCAGGGGCAAAC	CAAAGTGGCCAAGGTTCTCA
Bfsp1	GGAAATGCTGGAACGGCTTA	GAAACTGTGCCTCCAACTGAA
Bfsp2	TCCTGCTGCAGATGGAGAC	CTGAATGGCTGCTCGTTTTCA
Capn3	CCGTCTACAGCACCAGGTTA	CCAGCTTCGTGAAATGGTAGAC
Cd24a	GCTGGGGTTGCTGCTTC	GGAAACGGTGCAACAGATGT
Cd48	GTCTGGTCCTGGAACTGCTAC	TGCCGGTGGTGGCATTTATA
Cd63	CCCTGGGCATTGCTTTTGT	CACTTCGAATACTCTTCACCAGAC
Cd81	GCTGTACCTGGAACTGGGAA	AGCTCCCACAGCAATGAGAA
Cd84	TGGATCTGGTTCCTTTGCCTA	ACTGACTCCCCAAGAATCCC
Cd9	TGTGGAGCTGTACAAGAGTCC	TGGCGAATATCACCAAGAGGAA
Cpe	AAGTGGCAGTTCCTTTTAGCC	CCTCCTCCTTCCTTTCAGAGAA
Cryaa	GGAGATTCACGGCAAACACA	GTCCACATTGGAAGGCAGAC
Cryab	CACGGCAAGCACGAAGAAC	ATCCGGTACTTCCTGTGGAAC
Cryba1	AGCCATGGGTTGGTTCAACA	ATCCAGGGTACTGGTAGCAAAC
Cryba2	TGTGGGTTCCCTGAAAGTCA	ATACTGGTAACCTCGGTAGCC
Cryba4	GAGAGGCTCACCTCCTTCC	AGGAAGTTCTCCTGCTCGAA
Crybb1	ACCGGCTCATGTCCTTCC	TCCATGGTGTTGCCCTTGAA
Crybb2	AGCTCTCTGAGGCCCATCAA	GCCAGTAAAGTTGGGGTTCTCA
Crybb3	AGAAGGTGGGCTCCATCCAA	AACAAACTGCTCCCCACGAAA
Crybg3	CATCGGATCGATTCGTGTCA	AGGAATTGCTGGCCTTTGAA
Cryga	GATGGGTTTCAGCGACTCCA	CCCCGGTAGTCATCTCTCTCA
Crygb	CAGCGACTCCATTCGTTCC	TGAAGTGGAAGCGATCCTGAA
Crygc	CCCACAGAATGCGGCTGTA	TCGCTCAGCTCCATCATGAC
Crygd	CAGTGGATGGGTTTCAGTGAC	CTGGCCTCTGTACTCTTCCC
Cryge	AGGCCAAATGGTGGAGATCA	ATCACGTGGAAGGAGTGGAA
Crygf	CCAGCAGTGGATGGGTTTCA	CTCGCTCGTAGATCCTGATCC

Crygn	AGCTGTGTCAACGCCATCA	GCGGTAGTTTGGCTCCTCATA
Crygs	CGTTGGATGGGCCTTAATGAC	CGTTGAAGTCGCCCTTTTCA
Ctnnb1	CATTGGTGCCCAGGGAGAA	GCCGTATCCACCAGAGTGAAA
Dnase2b	GGTGTCCCTGGATCTGTGAA	CCCTGCGTTCTGTTCCATAC
Eef1a1	GTCGCCTTGGACGTTCTTTT	GCTTTGAATTAGCGGTGGTTTTC
Eftud2	CGGACACCAAAGGGAAGTCTTA	CCAGCTGTGACCTCATCAGAA
Eno1	CGCCTGGCCAAGTACAATCA	CTGAAGGACCTGCCAGCAAA
Fabp5	TTGGTTTACCCAGGATCATTCC	CCTTGAAGAACCCACACACA
Fgf2	TCTTCCTGCGCATCCATCC	GCACACACTCCCTTGATAGACA
Fgf5	ACGTCTCCACCCACTTCCTA	TTCTGGAACAGTGACGGTGAA
Fgf7	GGACCCAGGAGATGAAGAACA	CACCCCTTTGATTGCCACAA
eGFP	GCTGACCCTGAAGTTCATCT	GACTTGAAGAAGTCGTGCTG
Gapdh	AGACGGCCGCATCTTCTT	TTCACACCGACCTTCACCAT
Glud1	CTGACGTGAGTGTGGATGAA	TTAGCACCTCCAAACGGTAC
Golga2	TCTTGGTGTCACCCCTTCC	CTCCTCCATGAGGTTAGAGCAA
Grifin	AGGAGGAGGTGTCCAGCATA	TGTTCTGCGTCTGCACTCA
Gstp1	TCTACGCAGCACTGAATCC	CTCGAACTGGGAAGTAGACAA
Hif1a	TCGACACAGCCTCGATATGAA	TTCCGGCTCATAACCCATCA
Hsf1	ATGGACTCCAACCTGGACAA	AGGCTCTTGTGGAGACAGAA
Hsf2	GAAACTATTGAGTCCAGGCTTTCA	CTGGGCATGCTTTGCTCTTA
Hsf4	CTGTTTGGGCCACTTCAGAC	CGCTCCCCTCATCTAGCATTA
Hspa4	CTCGGCCTGTACACAGAGAA	AGCATTCTTGGCGTCATTCC
Hspb1	CGGAGATCACCATTCCGGTTA	TGGCTCCAGACTGTTCAGAC
Hspb2	TACTAGTCGCAACAGCAGTCA	TGGGGTTGGCAAATTCGTAC
Hspb6	CCAGTGTGGCGTTACCC	CTCTGGCAAGAAGTGCTTCA
Htra3	ACCATCCAGGACATCGACAA	TGAGTGACCCAGCAGCAA
Id2	ACCCTGAACACGGACATCA	TCGACATAAGCTCAGAAGGGAA
Igf2	GTCTACCTCTCAGGCCGTAC	GGACTGTCTCCAGGTGTCATA
Itgb1	AAGGGCCAACTTGTGAGACA	TGAAGGCTCTGCACTGAACA
Lamp1	GCTTTCAAGGTGGACAGTGAC	CCACAGCAATGGGGATCAAC
Lamp2	ACTACCTGTCTGCTGGCTAC	GTTGTGGCAGGGTTGATGTTA
Lenep	CCTCCAGGTGCCTGTCATTAA	GGATGTAGGCGACTTCCTTCA
Lim2	AACAAGTGCTTCCTGCAGAC	AGGGCAGACAGGATCATGAA
Maf1	TTCAGCACAGCCAGAAGTCA	TGAAAACAGGCTGCAGTTGAC
Map1lc3a	TGAGCGAGTTGGTCAAGATCA	TGGTTGACCAGCAGGAAGAA
Mcpt4	ACAAATCGTTCACCCAAAGTACA	GGACGAGGCAGAGGAATTACA
Mip	GCTGTTGGCTTCTCCCTCA	CATCCCCGCACCAGTGTAATA
Mmp2	CGAGGACTATGACCGGGATA	GGGCACCTTCTGAATTTCCA
Myl1	CCCACCAATGCAGAGGTCAA	AGCTTGCATCATGGGCAGAA
Nap1l4	AAGAAGGCGAGGAAGGTGAA	GGGGTTAACATCAGCATCATCC
Nupr1	AAGGTCGGACCAAGAGAGAA	AACTTGGTCAGCAGCTTCC
Opn1sw	AAGAGCTCCTGTGTCTACAACC	CCTGCACACCATCTCCAGAA
Pax6	GGGGTCTGTACCAACGATAACA	TCTGTTGCTTTTCGCTAGCC
Rab11a	AGGCACAGATATGGACACA	ATAAGGCACCTACTGCTCCA
Rab11fip1	TGCTCGGTCTCGATAAGTTCC	GGGTTTGGACTTCAGGGTGTA

Rab27a	TGCTTCTGTTCGACCTGACA	ACAGTACGCGTGCATCTGTA
Rab27b	CATCTGCAGCTTTGGGACAC	AAGCCCATGGCATCTCTGAA
Rab5a	CAGGAAACAAAGCTGACTTAGCA	TGATGTCTTAGCTGATGTCTCCA
Rab7	TGGACGACAGACTTGTTACCA	ACCTCTGTAGAAGGCCACAC
Rnaset2b	GGCGTGGCTTAGGGGTTA	ACCTCCACCGACTCCTCA
Rpl41	ACGCCATTAAATAGCAGTAGGC	TCTCATGGCGCAGGAGT
Serpinh1	GATGGGGCACTGCTTGTGAA	TAGGAGCGGGTCACCATGAA
Slamf8	GGTCAAACCTGGACCCAGAC	GCAGCAGTGAACACTTGAACC
Sox2	CCTGCAGTACAACTCCATGAC	TGCGAGTAGGACATGCTGTA
Sparc	GAAACCGTGGTGGAGGAGAC	TGCACCGTCCTCAAATTCTCC
Srsf2	CAAGAGCCCACCCAAGTCT	GTTAAGCCGCTTGCCGATT
Tdrd7	GTTCTGCTCGCTTTCCTTTCA	TTCACAAGGTCAGGGTCATCA
Tgfb2	GCCCATATCTATGGAGTTCAGACA	AGCGGAAGCTTCGGGATTTA
Tuba1a	AGTGTTCGTAGACCTGGAACC	AGTGGCCACGAGCATAGTTA
Uba52	CGAGAATGTCAAGGCCAAGATC	CTGTTTGCCCGCGAATATCA
Ubb	ATTCGGTCTGCATTCCCAGT	AATTGGGGCAAGTGGCTAGA
Vim	GATTTCTCTGCCTCTGCCAAC	CAACCAGAGGAAGTGACTCCA

****
